# Natural Killer Cells Degenerate Intact Sensory Afferents following Nerve Injury

**DOI:** 10.1016/j.cell.2018.12.022

**Published:** 2019-02-07

**Authors:** Alexander J. Davies, Hyoung Woo Kim, Rafael Gonzalez-Cano, Jahyang Choi, Seung Keun Back, Seung Eon Roh, Errin Johnson, Melanie Gabriac, Mi-Sun Kim, Jaehee Lee, Jeong Eun Lee, Yun Sook Kim, Yong Chul Bae, Sang Jeong Kim, Kyung-Mi Lee, Heung Sik Na, Priscilla Riva, Alban Latremoliere, Simon Rinaldi, Sophie Ugolini, Michael Costigan, Seog Bae Oh

**Affiliations:** 1 Dental Research Institute and Department of Neurobiology and Physiology, School of Dentistry, Seoul National University, Seoul 03080, Republic of Korea; 2 Department of Brain and Cognitive Sciences, College of Natural Sciences, Seoul National University, Seoul 08826, Republic of Korea; 3 Departments of Anesthesia and Neurobiology, Children’s Hospital Boston and Harvard Medical School, Boston, MA 02115, USA; 4 Departments of Physiology, Biochemistry and Molecular Biology, College of Medicine, Korea University, Seoul 02841, Republic of Korea; 5 Department of Physiology, Seoul National University College of Medicine, Seoul 03087, Republic of Korea; 6 Sir William Dunn School of Pathology, University of Oxford, South Parks Road, Oxford OX1 3RE, UK; 7 Aix Marseille Univ, CNRS, INSERM, CIML, Centre d’Immunologie de Marseille-Luminy, Marseille, France; 8 Department of Anatomy and Neurobiology, School of Dentistry, Kyungpook National University, Daegu 700-412, Korea; 9 Neurosurgery Department, Johns Hopkins School of Medicine, Baltimore, MD 21287, USA; 10 Nuffield Department of Clinical Neurosciences, University of Oxford, John Radcliffe Hospital, Oxford OX3 9DU, UK

**Keywords:** autoimmunity, dorsal root ganglia, innate immunity, natural cytotoxicity, neurodegeneration, neuroimmune, neuropathic pain, peripheral neuropathy, sciatic nerve crush, Wallerian degeneration

## Abstract

Sensory axons degenerate following separation from their cell body, but partial injury to peripheral nerves may leave the integrity of damaged axons preserved. We show that an endogenous ligand for the natural killer (NK) cell receptor NKG2D, Retinoic Acid Early 1 (RAE1), is re-expressed in adult dorsal root ganglion neurons following peripheral nerve injury, triggering selective degeneration of injured axons. Infiltration of cytotoxic NK cells into the sciatic nerve by extravasation occurs within 3 days following crush injury. Using a combination of genetic cell ablation and cytokine-antibody complex stimulation, we show that NK cell function correlates with loss of sensation due to degeneration of injured afferents and reduced incidence of post-injury hypersensitivity. This neuro-immune mechanism of selective NK cell-mediated degeneration of damaged but intact sensory axons complements Wallerian degeneration and suggests the therapeutic potential of modulating NK cell function to resolve painful neuropathy through the clearance of partially damaged nerves.

## Highlights

• Cytotoxic NK cells infiltrate the damaged peripheral nerve within days of injury
• Injured sensory axons express NKG2D ligand RAE1 to signal degeneration by NK cells
• Clearance of damaged axons reduces development of chronic pain after nerve injury
• NK cells complement Wallerian degeneration to aid functional regeneration of PNS

## Graphical Abstract

**Figure:**
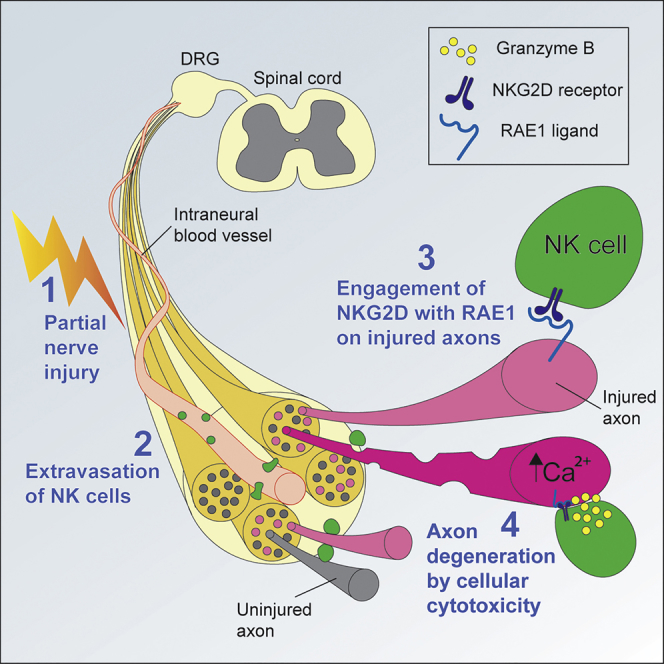

## Teaser

Pain following nerve damage can be mitigated by cytotoxic natural killer cells that selectively clear out partially damaged nerves.

## Introduction

Injury to a peripheral nerve results in the destruction of severed axons distal to the injury site via Wallerian degeneration (Conforti et al., 2014) thereby facilitating subsequent debris removal by immune cells (Dekkers et al., 2013). Simultaneously, a genetic program of axonal outgrowth is then initiated to allow the re-innervation of targets by healthy nerves (Chandran et al., 2016), which is in turn supported by the permissive environment created by infiltrating immune cells (Gaudet et al., 2011). While this adaptive response to axonal injury facilitates the phagocytosis of debris and ultimately regeneration of axons, it also contributes to the maladaptive symptom of neuropathic pain (Costigan et al., 2009). As a consequence, neuro-immune signaling mechanisms of hypersensitivity following injury to the adult peripheral nervous system (PNS) are the subject of intense research (Chavan et al., 2017).

Axon degeneration is generally regarded as neuron autonomous; however, discrepancies in the timing of axon degeneration after injury (Neukomm and Freeman, 2014) and recent evidence pointing to the role of as-yet-unknown extrinsic factors in axon fragmentation (Gamage et al., 2017) open up the possibility of a role for immune cells with cytotoxic action in the degeneration process itself.

Natural killer (NK) cells are cytotoxic innate lymphoid cells (ILCs) typically involved in the destruction of tumor or virus-infected target cells. NK cell activity is coordinated by a balance of stimulatory and inhibitory signals mediated via a distinct receptor repertoire (Vivier et al., 2011). One of the best-characterized activating receptors is NK group 2D (NKG2D), which is expressed on NK cells and some T cells. Endogenous ligands for NKG2D are normally absent from most tissues but are expressed in disease states such as tumorigenesis and infection (Raulet et al., 2013). NK cells are found among the immune cells that infiltrate the damaged nerve following injury in adult animals (Cui et al., 2000), and the presence of intra-neural NK cells correlates with disease severity in a form of peripheral neuropathy (Lackowski et al., 1998). NK cells have previously been shown to be involved in guanethidine toxicity to sympathetic neurons (Hickey et al., 1992). In addition, adult murine NK cells are directly cytotoxic to immature embryonic dorsal root ganglion (DRG) neurons *in vitro* (Backström et al., 2000). However, NK cells at this stage of primary sensory neuron development appear to be immature (Tang et al., 2012), and thus the consequences of this NK cell interaction for the adult are unclear.

We investigated the response and functional consequences of NK cell interactions in the context of peripheral nerve injury in the adult mouse. Our results identify cytotoxic NK cells as a novel cellular trigger for the specific degeneration of damaged primary afferent axons that are still connected to their cell bodies in the injured PNS. Enhancing this NK cell-dependent degeneration mechanism increased clearance of damaged axons resulting in lower levels of mechanical hypersensitivity post-injury. This discovery provides new insight into immune mechanisms of damaged axon clearance and suggests the therapeutic potential of interventions that modulate NK cell function.

## Results

### Activated NK Cells Induce Cytotoxicity in Embryonic Sensory Neurons by an RAE1-Mediated Mechanism

**Figure S1:**
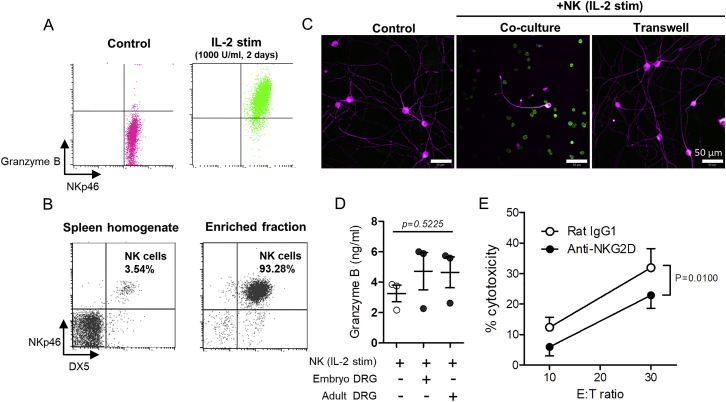
Isolation, Enrichment, and Activation of NK Cells from Adult Mouse Spleen and Cytotoxicity of IL-2-Stimulated NK Cells against Embryonic DRG Neurons, Related to Figure 1 (A) Intracellular staining of granzyme B of freshly isolated (*left*) and IL-2 stimulated (*righ*t) NK cells. (B) Flow cytometry plot of splenic lymphocytes. NKp46+DX5+ NK cells (top-right quadrant) in whole spleen homogenate (*left*) and after MACS enrichment by negative selection (*right*). NK cells require cell contact to mediate toxicity against embryonic DRG neurons. (C) Representative immunostaining images of embryonic DRG neurons (β-tubulin III, magenta) after 4 h co-culture with IL-2 stimulated NK cells (NKp46, green) either in direct contact or separated by a transwell membrane (0.4 μm pores). (D) ELISA detection of granzyme B in media of NK cell-DRG co-cultures. One way ANOVA, F(2,6) = 0.7248, p = 0.5225. n = 3 experimental repeats. Granzyme B was not detected in cultures containing unstimulated NK cells (data not shown). (E) NKG2D receptor contributes to NK cell-mediated lysis of embryonic DRG neurons. Cytotoxicity of IL-2 stimulated NK cells against embryonic DRG neurons after 4 h co-culture in the presence of blocking anti-NKG2D antibody or IgG control antibody. Two-way ANOVA: Effect of antibody treatment, F(1,4) = 21.21, p = 0.0100. n = 3 experimental repeats.

**Figure 1:**
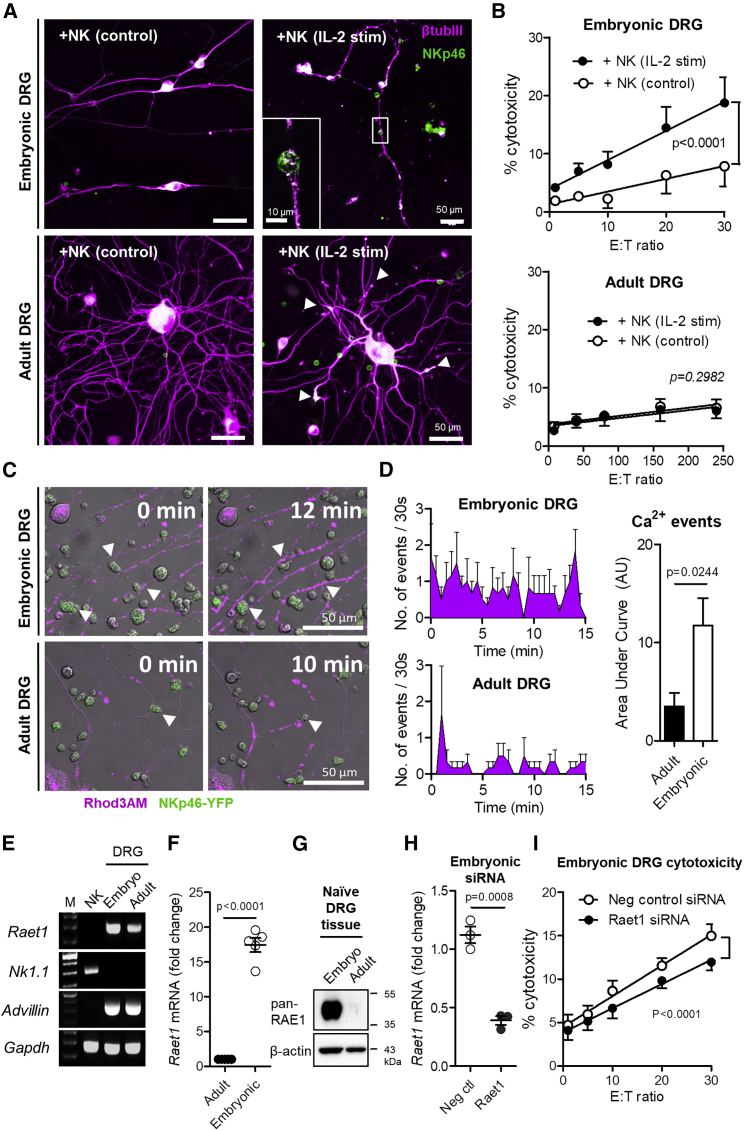
Acutely Cultured Embryonic but Not Adult DRG Neurons Reveal Susceptibility to NK-Mediated Cytotoxicity by RAE1 (A) Immunolabeling of co-culture (4 h) between embryonic (top) or adult (bottom) DRG neurons (β-tubulin, magenta) and either freshly isolated (control) or IL-2-stimulated natural killer (NK) cells (NKp46, green). The inset shows a high-magnification image of NK cell in contact with embryonic DRG neurite. (B) LDH-release cytotoxicity assay of acutely cultured (1 day *in vitro*) embryonic (top) and adult (bottom) DRG at various Effector (NK):Target (DRG) (E:T) ratios. Matched two-way ANOVA: embryonic DRG, F(1,10) = 100.01, p < 0.0001); adult DRG, F(1,10) = 1.25, p = 0.2982). Three replicate co-cultures for each DRG group. (C) Still images of *in vitro* time-lapse confocal Ca^2+^ imaging of rhodamine 3 AM-loaded embryonic (top) and adult (bottom) DRG (magenta) co-cultured with IL-2-stimulated NK cells (green) isolated from adult male NKp46-YFP mice. (D) Frequency histogram (30 s time bins) of neurite Ca^2+^ events in embryonic (top) and adult (bottom) DRG during NK co-culture. Cumulative area under the curve (right). Student’s paired t test; t = 2.290, p = 0.045. n = 6 fields of view from two repeat co-cultures per group. (E) RT-PCR of mRNA transcripts in freshly isolated splenic NK cells and embryonic and adult DRG. (F) qRT-PCR shows higher *Raet1* mRNA expression in embryonic compared to adult DRG tissue. Student’s paired t test; t = 16.16, p < 0.0001. n = 5 mice, or replicates per group. (G) Western blot of embryonic and adult mouse DRG tissue (40 μg loading) with pan-RAE1 antibody and β-actin control. Images are representative of three independent experiments. (H) Selective siRNA knockdown reduces RAE1 protein (top) and *Raet1* mRNA (bottom) expression in embryonic DRG (2 d culture). Student’s unpaired t test; t = 9.060, p = 0.0008. n = 3 mice, or replicates per group. (I) LDH-release cytotoxicity assay of negative control or *Raet1*-selective siRNA knockdown embryonic DRG. Three replicate co-cultures for each siRNA group. Matched two-way ANOVA F(1,10) = 133.85, p < 0.0001). See also Figure S1.

NK cells are classically activated by the cytokine interleukin (IL)-2, which primes them for cytotoxic attack by increasing intracellular content of granzyme B (Figure S1A). We examined the effects of IL-2-stimulated NK cells on DRG neurons acutely isolated from embryonic (E15) and adult mice (cultured less than 24 h *in vitro*), using freshly isolated, unstimulated splenic NK cells as a control (Figure S1B). Confirming original reports (Backström et al., 2000), embryonic DRG neurons were highly susceptible to NK cell-mediated cytotoxicity (Figures 1A and 1B). Separating NK cells and embryonic DRG with a transwell membrane prevented neurite fragmentation (Figure S1C) despite the presence of nanogram amounts of granzyme B in the culture media (Figure S1D). In contrast, we found no evidence of cell lysis of acutely isolated adult DRG neurons by lactate dehydrogenase (LDH)-release cytotoxicity assay, even at much higher effector-to-target (E:T) ratios (Figure 1B), although some neurites appeared truncated (Figure 1A).

Time-lapse confocal imaging of DiI-labeled DRG neurons co-cultured with IL-2-stimulated NK cells expressing yellow fluorescent protein (YFP) revealed that stimulated NK cells were highly motile, enabling multiple direct cell-cell contacts between NK cells and sensory neurons. In embryonic DRG, this led to neurite destruction and cell death (Video S1); however, acutely cultured adult DRG neurite membranes remained largely intact (Video S2). NK cell contacts were often synchronous with intracellular Ca^2+^ events in embryonic DRG neurites (Figures 1C and 1D; Video S3), leading to neurite fragmentation. While Ca^2+^ events were also observed in adult DRG after NK cell contact (Figures 1C and 1D) and occasionally led to neurite fragmentation (Video S4), they occurred at a much lower frequency than in embryonic neurons (Figure 1D).

Video S1. Live Confocal Imaging of Embryonic DRG Neuron Cytotoxicity by IL-2-Stimulated NK Cells *In Vitro*, Related to Figure 1

Video S2. Live Confocal Imaging of Adult DRG Neuron Resistance to Toxicity by IL-2-Stimulated NK Cells *In Vitro*, Related to Figure 1

Video S3. Live Confocal Ca^2+^ Imaging of Embryonic DRG Neuron Cytotoxicity by IL-2-Stimulated NK Cells *In Vitro*, Related to Figure 1

Video S4. Live Confocal Ca^2+^ Imaging of Adult DRG Neuron Undergoing Local Axon Degeneration by IL-2-Stimulated NK Cells *In Vitro*, Related to Figure 1

Retinoic Acid Early inducible protein 1 (RAE1), encoded by the *Raet1* gene family (α,β,γ,δ,ε), acts as a membrane-bound ligand for the activating receptor NKG2D (Cerwenka et al., 2000), which has previously been implicated in NK cell-mediated lysis of embryonic DRG (Backström et al., 2003). First, we confirmed that NK cytotoxicity against embryonic DRG could be attenuated by an NKG2D receptor blocking antibody (Figure S1E). Using universal primers, we observed *Raet1* transcripts in acutely dissociated embryonic and adult DRG neurons (Figure 1E), but they were 17 times more abundant in embryonic DRG relative to adult DRG when assayed by qPCR (Figure 1F). Western blot using a pan-RAE1 antibody revealed a band at approximately 40–50 kDa in embryonic but not adult DRG tissue (Figure 1G). To assess the functional contribution of *Raet1* in embryonic DRG neurons, we selectively knocked down all *Raet1* isoforms using small interfering RNA (siRNA) (Figure 1H), which, compared to negative control, led to a 20% reduction in NK-mediated lysis (Figure 1I).

### Nerve Injury Drives RAE1 Expression in Adult Sensory Neurons, Allowing Cytotoxic Attack by Activated NK Cells

**Figure 2:**
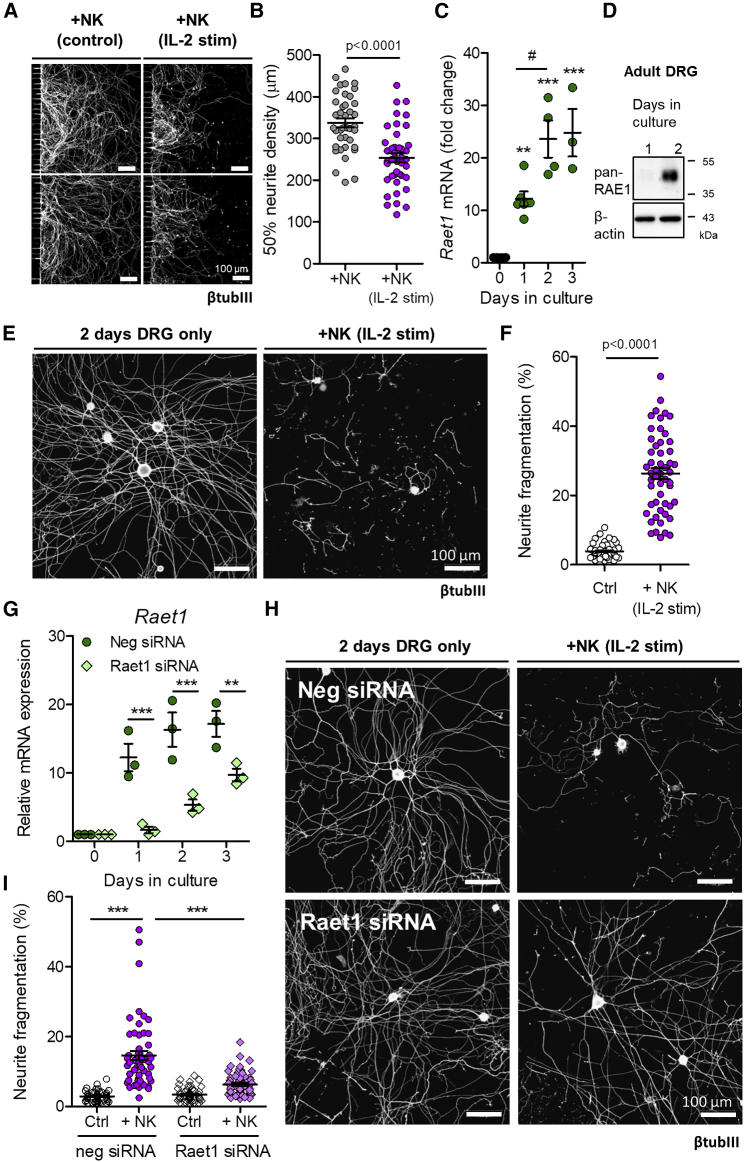
*Raet1* Expression Is Upregulated in Dissociated Adult DRG *In Vitro* and Confers NK Cell-Mediated Neurite Fragmentation (A) Microfluidic culture of adult DRG (5 days *in vitro*) exposed to freshly isolated (control) or IL-2-stimulated NK cells (4 h) in the neurite compartment immunolabeled with β-tubulin III. (B) Quantification of DRG neurite density. n = 6–7 regions per microfluidic device, n = 3 devices per group, two independent experimental repeats. Student’s unpaired t test; t = 5.448, p < 0.0001. (C) *Raet1* mRNA expression in adult DRG cultures by qPCR. One-way ANOVA; F (3,15) = 25.94, ^∗∗∗^p < 0.0001 with Bonferroni post-test: #p < 0.05, ^∗∗^p < 0.001, ^∗∗∗^p < 0.0001; n = 3–6 mice, or replicate cultures per time point. (D) RAE1 protein expression in adult DRG cultures 1 and 2 days *in vitro* (representative of three independent experiments). 25 μg protein loading. (E) Adult DRG culture (2 days *in vitro*) alone or co-cultured with IL-2-stimulated NK cells immunolabeled with β-tubulin III. (F) Quantification of DRG neurite fragmentation. Student’s unpaired t test; t = 12.74, p < 0.0001. n = 6–8 regions per coverslip, n = 3 coverslips per group, two independent experimental repeats. (G) siRNA knockdown of *Raet1* mRNA expression in adult DRG *in vitro*. Two-way ANOVA: effect of siRNA, F(1,16) = 54.02, ^∗∗∗^p < 0.0001 with Bonferroni post-test: ^∗∗^p < 0.001, ^∗∗∗^p < 0.0001. n = 3–5 cultures per group. (H) siRNA-transfected adult DRG culture (2 days *in vitro*) alone or co-cultured with IL-2-stimulated NK cells (4 h) immunolabeled with β-tubulin III. (I) Quantification of DRG neurite fragmentation. One-way ANOVA; F (3,201) = 51.16, p < 0.0001 with Bonferroni post-test: ^∗∗∗^p < 0.0001; n = 6–8 regions per coverslip, n = 6 coverslips per group, two independent experimental repeats. See also Figure S2.

**Figure S2:**
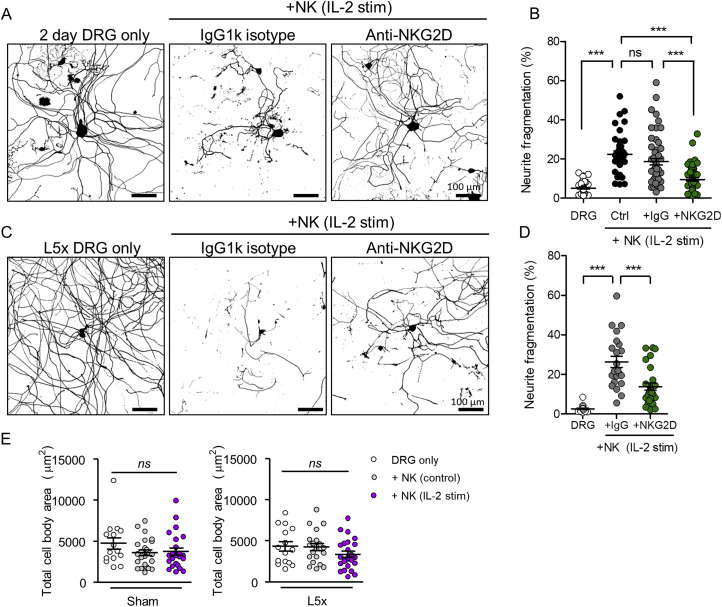
Antibody Blockade of NKG2D Receptor on NK Cells Attenuates Neurite Degeneration of Adult DRG Neurons *In Vitro*, Related to Figures 2 and 3 (A) Immunolabeling (β-tubulin III) of cultured adult DRG (10^3^ cells per coverslip) (2 days *in vitro*) co-cultured (4 h) with IL-2 stimulated NK cells (2.5x10^5^ cells per coverslip) pre-treated with either anti-NKG2D or IgG isotype control antibody. (B) Quantification of DRG neurite fragmentation after NKG2D receptor blockade. One way ANOVA, F(3,193) = 36.82, p < 0.0001 with Bonferroni post-test, ^∗∗∗^p < 0.001. n = 6-8 regions per coverslip, n = 4-6 coverslips per group, two independent experimental repeats. (C) L5 DRG neurons were cultured 7 days after L5x injury (24 h in culture) and exposed to IL-2 stimulated NK cells in the presence of anti-NKG2D (CX5) blocking antibody or IgG1k isotype control (30 μg/ml) for 4 h. Images representative of β tubulin III labeling of fixed cultures in each condition. (D) Quantification of DRG neurite fragmentation in acute (< 24 h) cultures of L5x injured DRG neuron-NK co-cultures. One-way ANOVA; F (2,67) = 32.07, p < 0.0001 with Bonferroni post-test:: t = 7.999, t = 4.492; ^∗∗∗^p < 0.0001; n = 6-8 regions per coverslip, n = 3 coverslips per group. (E) Quantification of total DRG cell body area in acute (< 24 h) cultures of sham and L5x injured DRG neuron co-cultured with or without control or IL-2 stimulated NK cells (4 h). One-way ANOVA: Sham, F(2,61) = 1.457, p = 0.2409; L5x, F(2,56) = 1.559, p = 0.2194. Bonferroni post-tests: ns, p > 0.05. n = 6-8 regions per coverslip, n = 3-6 coverslips per group, two independent experiments.

Adult mouse DRG neurons were cultured in a microfluidic chamber for 5 days *in vitro*. Following exposure to stimulated NK cells, we saw a 25% loss of neurite coverage relative to freshly isolated control NK cells (Figures 2A and 2B). *Raet1* mRNA was time-dependently upregulated in adult DRG cultures (Figure 2C), with corresponding *de novo* expression of RAE1 protein after 2 days *in vitro* (Figure 2D). Subsequently, adult DRG neurons cultured for 2 days displayed over a 10-fold increase in neurite fragmentation relative to controls in the presence of stimulated NK cells (Figures 2E and 2F). Transfection of dissociated adult DRG neurons with *Raet1* siRNA prior to culture delayed *Raet1* upregulation (Figure 2G) and reduced the ability of stimulated NK cells to fragment DRG neurites relative to negative control siRNA (Figures 2H and 2I). We also confirmed that fragmentation of adult DRG neurites (2 days *in vitro*) can be reduced by blocking the NKG2D receptor on NK cells prior to co-culture (Figures S2A and S2B).

**Figure 3:**
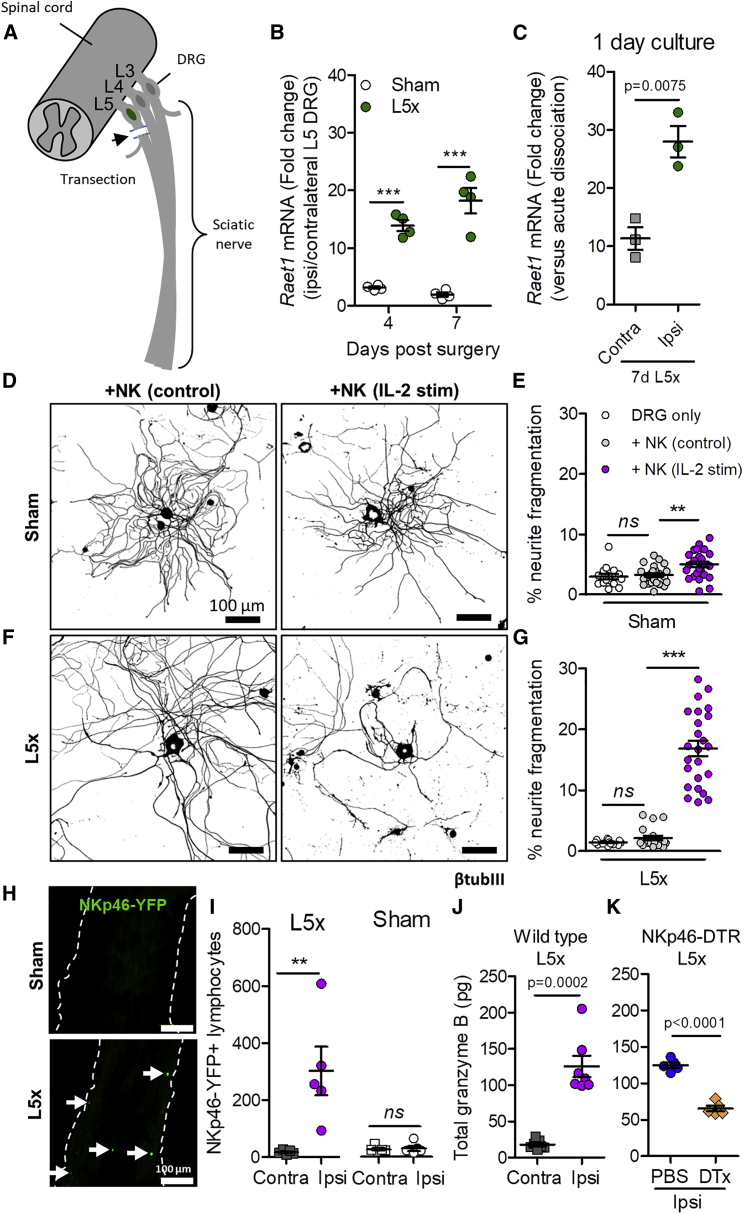
Peripheral Nerve Injury Regulates Raet1 Expression and Injured Sensory Neurons Show Increased Neurite Fragmentation by Stimulated NK Cells (A) Schematic diagram of spinal nerve transection injury site in relation to lumbar DRGs (L3, L4, and L5) (B) *Raet1* mRNA expression in ipsilateral L5 DRG after spinal nerve transection relative to contralateral DRG by qPCR. Two-way ANOVA: effect of injury, F(1,12) = 121.25, p = 0001; effect of time, F(1,12) = 5.08, p = 0.0438. Bonferroni post-test, ipsi versus contra: 4 days, t = 6.193, ^∗∗∗^p < 0.001; 7 days, t = 9.380, ^∗∗∗^p < 0.001. n = 4 samples per time point, n = 2 mice DRG pooled per sample. (C) qPCR shows injury-related increase in *Raet1* mRNA expression in adult DRG (1 day *in vitro*). Student’s unpaired t test, t = 4.994, p = 0.0075. n = 3 mice, 3 replicate cultures. (D) Immunolabeling (β-tubulin III) of cultured L5 DRG (1 day *in vitro*) isolated 7 d after sham surgery co-cultured (4 h) with either freshly isolated (control) or IL-2-stimulated NK cells. (E) Quantification of sham DRG neurite fragmentation. One-way ANOVA: F(2,61) = 7.171 p = 0.0016. Bonferroni post-test: DRG only versus control NK, t = 0.4349, nonsignificant (*ns*) p > 0.05; control NK versus IL-2-stimulated NK, t = 3.221 ^∗∗^p < 0.01; DRG only versus IL-2-stimulated NK, t = 3.180, ^∗∗^p < 0.01. (F) Immunolabeling (β-tubulin III) of cultured L5 DRG (1 day *in vitro*) isolated 7 days after L5 spinal nerve transection co-cultured (4 h) with either freshly isolated (control) or IL-2-stimulated NK cells. (G) Quantification of L5x DRG neurite fragmentation. One-way ANOVA: F(2,56) = 95.92, p < 0.0001. Bonferroni post-test: DRG only versus control NK, t = 0.5204, *ns* p > 0.05; control NK versus IL-2-stimulated NK, t = 11.45, ^∗∗∗^p < 0.001; DRG only versus IL-2-stimulated NK, ^∗∗∗^t = 11.86, p < 0.001. n = 6–8 regions per coverslip, n = 6 coverslips per group, two independent experimental repeats. (H) Sciatic nerve tissue sections from adult male NKp46-YFP mice 7 days after L5 spinal nerve transection injury immunolabeled with anti-GFP (NKp46, green). Arrows indicate NK cells in sciatic nerve. (I) Quantification of YFP^+^ events within lymphocyte gate from whole sciatic nerve homogenates. One-way ANOVA: F(3,16) = 10.61, p = 0.0004. Bonferroni post-test: L5x ipsi versus contra, t = 4.710, ^∗∗^p < 0.01; sham ipsi versus contra, t = 0.053, ns p > 0.05; L5x ipsi versus sham ipsi, t = 4.526, ^∗∗^p < 0.01 (n = 5 mice for each group). (J) ELISA quantification of granzyme B content in wild-type whole sciatic nerve after L5x injury. Student’s paired two-tailed t test: 7 days ipsi versus contra L5x, t = 8.088, p = 0.0002 (n = 7 mice). (K) ELISA quantification of granzyme B content in NKp46-DTR mice whole sciatic nerve after L5x injury. Student’s unpaired one-tailed t test: 7 days PBS versus DTx L5x ipsi, t = 11.03, p < 0.0001 (n = 5 mice per treatment). See also Figures S2 and S3.

We next asked whether *Raet1* upregulation in dissociated DRG *in vitro* occurs in injured cells *in vivo* by cutting the spinal nerve of the fifth lumbar DRG (L5x) in adult mice (Figure 3A). An injury-dependent upregulation of *Raet1* was revealed by qPCR using RNA isolated from L5 DRG at 4 and 7 days after L5x injury (Figure 3B). The difference in *Raet1* transcript levels between ipsilateral and contralateral L5 DRG 7 days after L5x injury was maintained over the first 24 h in culture (Figure 3C). Injured (L5x) DRG exhibited 3.5-fold more fragmentation of neurites when exposed to IL-2-stimulated NK cells (Figures 3F and 3G) than DRG from uninjured (sham) animals (Figures 3D and 3E), an effect that was attenuated by prior blocking of NKG2D receptors on NK cells (Figures S2C and S2D). No difference was seen in the total DRG cell body area between different groups (Figure S2E) suggesting that the fragmentation was neurite specific.

### Cytotoxic NK Cells Respond to Nerve Injury *In Vivo* and Target RAE1-Expressing Axons

**Figure S3:**
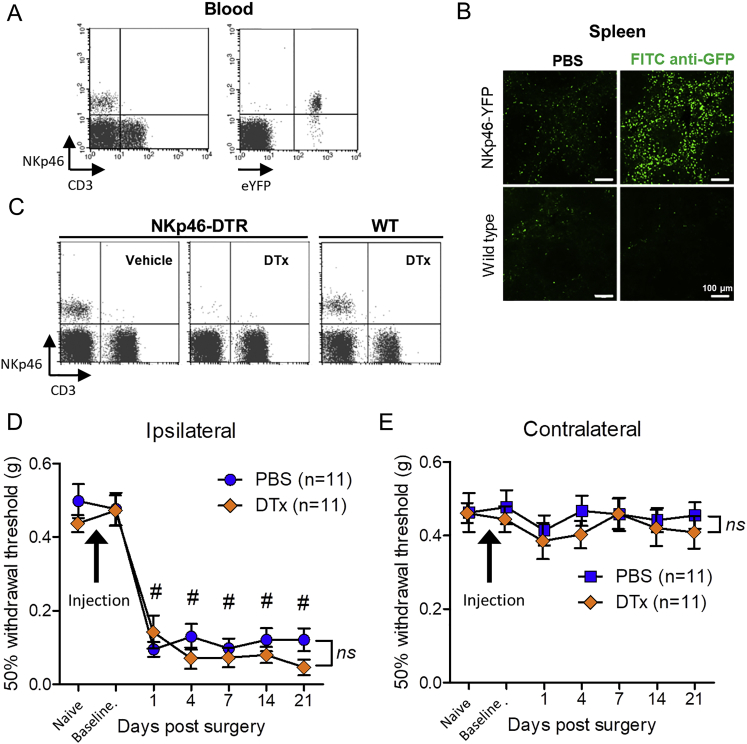
Characterization of NKp46-cre Mice, Related to Figures 3 and 4 (A) Flow cytometry of peripheral blood lymphocytes from NKp46-YFP mice labeled with anti-NKp46 and anti-CD3 antibodies. (B) Immunolabeling with an anti-GFP antibody (green) in spleen tissue sections from wild-type and NKp46-YFP mice. Note enhancement of signal only in labeled NKp46-YFP mouse spleen tissue. (C) Flow cytometry of peripheral blood lymphocytes from NKp46-DTR or wild-type (WT) mice 24 h after intravenous treatment with DTx (100 ng) or PBS vehicle (100 μl). Cells labeled with fluorescent-conjugated anti-NKp46 and anti-CD3 antibodies. (D) NKp46-DTR mice were chronically treated with DTx (n = 11) or PBS vehicle (n = 11) (i.v.) followed by L5 spinal nerve transection surgery (Day 0). Sensitivity to mechanical (von Frey filament) stimulation in the injured ipsilateral hind paw before (*naive*) and after injections (*baseline*) and at intervals after L5x injury. Ipsilateral: Two-way ANOVA (effect of time) F(6,140) = 62.24, #p < 0.0001; (effect of depletion) F(1,140) = 1.13, p = 0.2894 (*ns*, not significant). (E) Uninjured contralateral hind paw. Two-way ANOVA (effect of time) F(6,140) = 0.58, p = 0.7468; (effect of depletion) F(1,140) = 0.11, p = 0.7174.

To characterize the response and function of NK cells *in vivo*, we crossed *Ncr1*^*icre*^ mice (Narni-Mancinelli et al., 2011) with reporter mice that express either YFP (*Rosa26*^*eyfp*^) or the diphtheria toxin (DTx) receptor (*Rosa26*^*dtr*^; DTR) following Cre-mediated recombination. *Ncr1* encodes the NKp46 receptor, which is expressed on all NK cells, as well as tissue-resident ILCs, including group 1 ILCs (ILC1s) and a subset of group 3 ILCs (NCR1^+^ ILC3s). This genetic approach allowed us to identify (Figures S3A and S3B) or systemically deplete NKp46^+^ cells *in vivo* (Figure S3C). We did not observe YFP-positive NK cells in sciatic nerve in naive mice (data not shown) or sham surgery mice (Figures 3H and 3I). In contrast, L5x injury led to the marked recruitment of YFP^+^ NK cells to the injured sciatic nerve (Figures 3H and 3I) and was paralleled by an increase in granzyme B content at 7 days post-injury (Figure 3J), which was significantly reduced by prior systemic depletion of NK cells (Figure 3K). L5x injury led to a reduction in the mechanical withdrawal threshold in the ipsilateral hind paw of NKp46-DTR mice treated with PBS (Figure S3D) that was not affected by prior depletion of NK cells using DTx (Figure S3D). The sensitivity of the uninjured contralateral paw remained unaffected by either treatment (Figure S3E).

**Figure 4:**
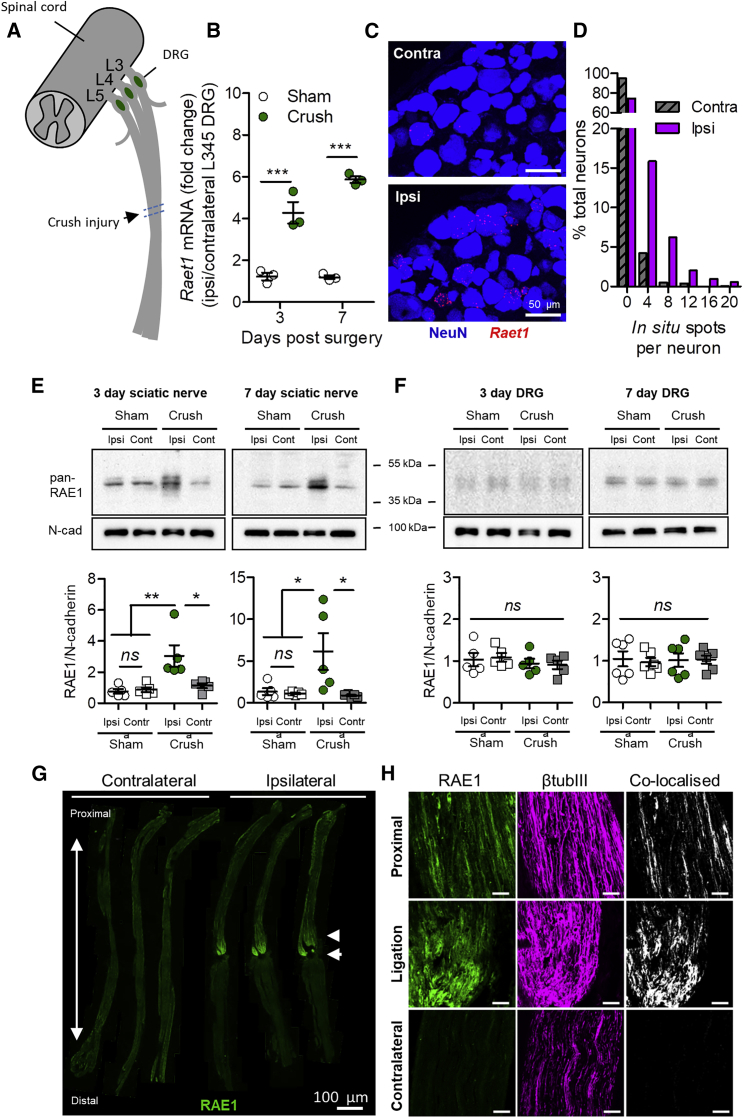
Sciatic Nerve Crush Increases RAE1 in Peripheral Nerve Axons (A) Schematic diagram of sciatic nerve crush injury site in relation to lumbar DRGs (L3, L4, and L5). (B) *Raet1* mRNA expression in ipsilateral L3–5 DRG 3 and 7 days after surgery by qPCR. Two-way ANOVA: effect of injury, F(1,8) = 180.95, p < 0.0001; effect of time, F(1,8) = 8.04, p = 0.0220. Bonferroni post-test: 3 days, t = 7.507, ^∗∗∗^p < 0.001; 7 days, t = 11.52, ^∗∗∗^p < 0.001. n = 3 mice per surgery per time point. (C) *In situ* hybridization with *Raet1* probe (red) in L4 DRG immunolabeled for NeuN (blue) 6 days after partial sciatic nerve crush injury. Scale bar, 50 μm. (D) Distribution of Raet1 *in situ* spots in L4 DRG ipsi and contralateral to sciatic crush. Kolmogorov-Smirnov (KS) test: ^∗∗∗^p < 0.0001, KS D-value 0.2297. n = 3 mice, n = 3 sections per ipsi and contralateral DRG per mouse. (E) (Top) Pan-RAE1 western blot of sciatic nerve tissue (40 μg protein loading) 3 days after sciatic nerve crush or sham surgery. Neuronal cadherin control is shown. (Bottom) Relative expression of RAE1 protein in ipsilateral versus contralateral sciatic nerves 3 and 7 days after sham or crush surgery. One-way ANOVA, 3 days: F(3,16) = 8.505, p = 0.0013. Bonferroni post-test: sham ipsi versus contra, t = 0.2025, ns p > 0.05; crush ipsi versus contra, t = 3.678, ^∗^p < 0.05; ipsi sham versus crush, t = 4.438, ^∗∗^p < 0.01. One-way ANOVA, 7 days: F(3,16) = 5.119, p = 0.0113. Bonferroni post-test: sham ipsi versus contra, t = 0.1438, ns p > 0.05; crush ipsi versus contra, t = 3.342, ^∗^p < 0.05; ipsi sham versus crush, t = 3.043, ^∗^p < 0.05 (n = 5 mice per surgery group per time point). (F) (Top) Pan-RAE1 western blot of DRG (L3–5) tissue (40 μg protein loading) 3 days after sciatic nerve crush or sham surgery. Neuronal cadherin control. (Bottom) Relative expression of RAE1 protein in ipsilateral versus contralateral DRG 3 and 7 days after sham or crush surgery. One-way ANOVA: 3 days, F(3,16) = 0.438, p = 0.7288; 7 days, F(3,20) = 0.053, p = 0.983 (n = 5–6 mice per surgery group per time point). (G) Composite images of RAE1 immunolabeling in full-length contralateral and ipsilateral sciatic nerves taken from three individual mice 3 days after tight sciatic nerve ligation. Arrow, ligation site; arrowhead, proximal to ligation. (H) Maximum projection images of RAE1 (green) co-localization with axonal marker β-tubulin III (magenta) in sciatic nerve 3 days after tight ligation. Scale bars, 50 μm. See also Figures S3 and S4.

**Figure S4:**
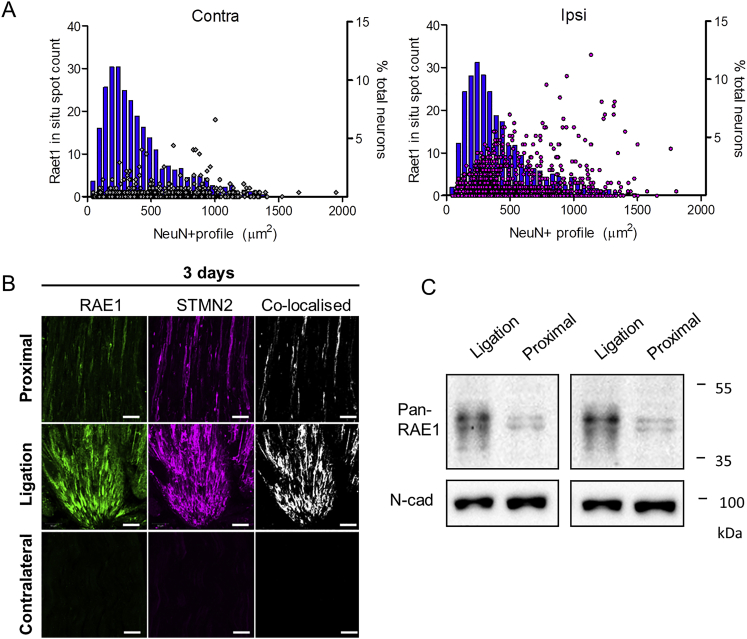
Injury-Dependent Regulation of *Raet1* mRNA and RAE1 Protein Expression in Sensory Neurons *In Vivo*, Related to Figure 4 (A) The size of all NeuN+ neuronal profiles in each DRG are shown as a histograms in blue (50 μm^2^ bins). The number of *in situ* ‘spots’ per NeuN+ neuronal profile are overlayed as a scatterplot. The majority of *de novo Raet1*-expressing NeuN+ profiles in ipsilateral DRG appear to be in the small-medium size range (200-500 μm^2^). A large number of *Raet1 in situ* spots are also seen NeuN+ profiles of 500 μm^2^ and greater in ipsilateral DRG. The overall size distribution of NeuN+ profiles was not different between DRG (Kolmogorov-Smirnov test: p = 0.0513, KS D-value = 0.04502). (B) Maximum projection images of RAE1 (*green*) co-localized with neuronal injury marker STMN2 (*magenta*) in sciatic nerve 3 days after tight ligation. Note lack of STMN2 immunolabeling in uninjured contralateral nerves. Scale bars, 50 μm. (C) western blots of ligated and proximal regions of sciatic nerve tissue 7 days after tight ligation (35 μg protein loading) with pan-RAE1 antibody and neuronal cadherin as loading controls. Blots from two independent experiments.

Consistent with the effect of nerve transection, crush injury (Figure 4A) also drove *Raet1* expression in ipsilateral DRG as observed by qPCR (Figure 4B). *In situ* hybridization revealed *Raet1* transcripts within individual DRG neurons (Figures 4C and 4D) and across a broad range of cell sizes (Figures S4A and S4B). RAE1 is known to be controlled by post-transcriptional modification (Raulet et al., 2013); therefore, we performed western blot on lysates of sciatic nerve and DRG tissues. Crush injury induced a 45-kDa band in ipsilateral injured sciatic nerve (Figure 4E), indicating an increase in the glycosylated mature form of RAE1 (Arapovic et al., 2009) in the nerve, but not in the DRG (Figure 4F). Chronic tight ligation of the sciatic nerve, to block axonal transport, revealed RAE1 immunolabeling within crushed axons proximal to the ligation site (Figures 4G, 4H, and S4B) and greater amounts of RAE1 protein at the ligation site relative to within the axons proximal to the ligation (Figure S4C).

**Figure S5:**
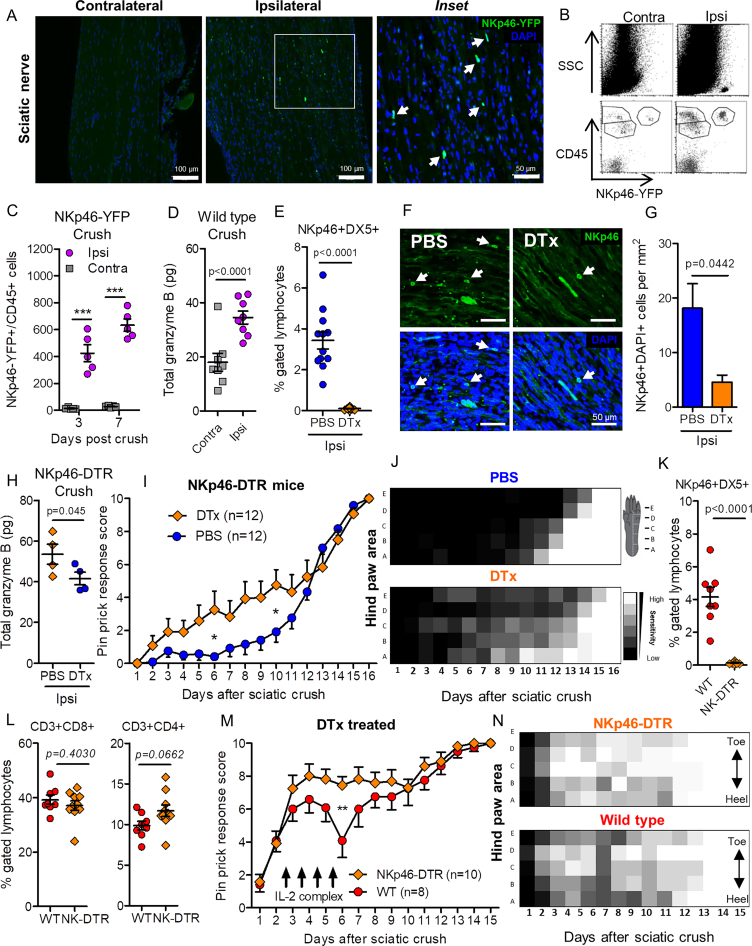
Systemic NK Cell Depletion Prevents Sensory Loss after Sciatic Nerve Crush, Related to Figure 5 (A) Sciatic nerve tissue sections from adult male NKp46-YFP mice 7 days after sciatic nerve crush injury. Inset shows higher magnification of ipsilateral nerve. Arrows indicate co-localization of NKp46-YFP (anti-GFP, *green*) and nuclear (DAPI, *blue*) labeling. (B) Flow cytometry scatterplots of sciatic nerve homogenates obtained from an NKp46-YFP mouse 3 days after sciatic nerve crush injury. (C) Quantification of total number of CD45+/YFP+ double-positive events within lymphocyte FSC/SSC gate from whole sciatic nerve homogenates. Two-way ANOVA, Crush: Effect of injury, F(1,16) = 168.63, p < 0.0001; Effect of time, F(1,16) = 8.10, p = 0.0117. Bonferroni post-test: 3 days ipsi versus contra crush, t = 7.426, ^∗∗∗^p < 0.001; 7 days ipsi versus contra crush, t = 10.94, ^∗∗∗^p < 0.001 (n = 5 mice per time point). (D) ELISA quantification of granzyme B content in whole sciatic nerve 7 days after crush injury in wild-type mice. ipsi *versus contra,* t = 7.112 (paired one-tailed t test, n = 8 mice); (E) NKp46+/DX5+ double-positive lymphocytes in peripheral blood 16 days after sciatic nerve crush. Student’s unpaired t test: t = 8.012, p < 0.0001. (F) NKp46 immunolabeling and nuclear (DAPI) staining in sciatic nerve sections (14 μm) 7 days after forceps crush injury in NKp46-DTR mice treated with DTx or PBS vehicle. (G) Quantification of NKp46+DAPI+ cells (white arrows) within a 0.4 mm^2^ region of the nerve crush site were counted by an investigator blind to the treatment. Student’s unpaired t test, t = 2.898, p = 0.0442. n = 2-3 sections per nerve, n = 3 mice per treatment. (H) ELISA quantification of granzyme B content in whole sciatic nerve 7 days after crush injury in NKp46-DTR mice after treatment with PBS or DTx. t = 2.024 (unpaired one-tailed t test, n = 4 mice per treatment). (I) Daily pinprick response score in NKp46-DTR mice treated intravenously with DTx or PBS vehicle every 4 to 5 days. Full sciatic nerve crush on day 0. Two-way ANOVA: Effect of depletion, F(1,352) = 25.20, p < 0.0001). n = 12 mice per group. Bonferroni post-test, ^∗^p < 0.05 (t = 3.048). (J) Heatmap showing mean sensitivity to pinprick along the lateral hind paw. (K) Peripheral blood sampled 16 days post-injury shows almost a complete loss of NKp46+DX5+ NK cells in DTx-treated NKp46-DTR mice compared to wild-type. Student’s unpaired t tests., t = 7.761, p < 0.0001. (L) CD3+CD8+ (t = 0.8590, p = 0.4030) and CD3+CD4+ (t = 1.971, p = 0.0662) T cell populations were not different between genotypes after DTx treatment (Student’s unpaired t tests). (M) 8 wild-type and 10 NKp46-DTR mice received partial crush of the sciatic nerve on day 0 as well as diphtheria toxin (DTx) i.v. starting one day before surgery and continuing every 4 to 5 days. All mice were treated with IL-2/anti-IL-2 antibody complex i.p. daily for four consecutive days from the evening of day 2 (arrows). Daily pinprick response shows a transient reduction in sensitivity in wild-type mice receiving the IL-2/anti-IL-2 antibody complex and DTx treatment but not NKp46-DTR mice. Two-way ANOVA. Effect of genotype: F(1,275) = 12.49, p = 0.0005). Bonferroni post-test, ^∗∗^p < 0.01 (t = 3.789). (N) Heatmap showing mean sensitivity to pinprick along the lateral hind paw.

Sciatic nerve crush recruited NKp46-YFP cells to the injury site (Figure S5A) and increased CD45^+^YFP^+^ lymphocyte-gated events within ipsilateral sciatic nerve (Figure S5B) by 30-fold 3 days after injury and a further 50% by day 7 (Figure S5C). Crush injury also raised levels of granzyme B in the sciatic nerve (Figure S5D). Using two-photon imaging of the sciatic nerve *in vivo* 3 days after crush injury, we observed NKp46-YFP cells “rolling” along the wall of intra-neural blood vessels (Video S5). Within the sciatic nerve itself, NK cells were highly motile and displayed multipolar morphology (Video S6), reminiscent of stimulated NK cells *in vitro*. No YFP^+^ cells were observed by two-photon imaging in the nerves of sham injured mice (data not shown).

**Figure 5:**
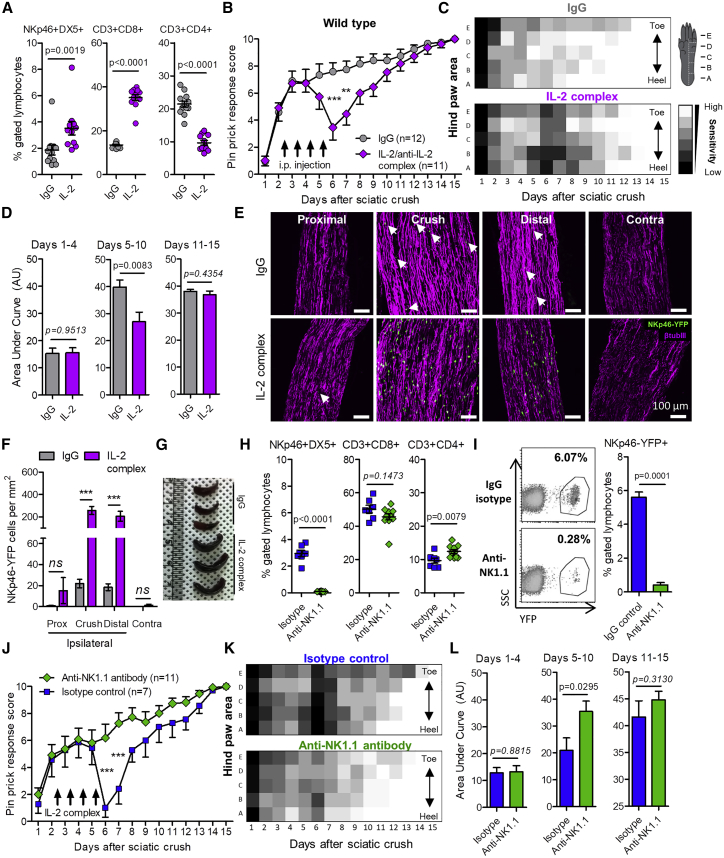
IL-2/Anti-IL-2 Antibody Complex Treatment Triggers NK Cell-Dependent Acute Sensory Loss after Partial Sciatic Nerve Crush (A) Peripheral blood sampled 16 days post-injury in IL-2 complex or IgG control mice. NKp46^+^DX5^+^ NK cells (U = 15.00, p = 0.0019); CD3^+^CD8^+^ T cells (t = 15.78, p < 0.0001); CD3^+^CD4^+^ T cells (t = 9.719, p < 0.0001). Mann-Whitney or Student’s unpaired t test was used. (B) Daily pinprick response. Male wild-type C57BL/6 mice received partial crush of the sciatic nerve on day 0 followed by daily injection of IL-2 complex or IgG control (i.p.) for 4 consecutive days (arrows). Two-way ANOVA was used. Effect of treatment: F(1,315) = 20.69, p < 0.0001. Bonferroni post-test ^∗∗^p < 0.01 (t = 3.784), ^∗∗∗^p < 0.001 (t = 4.741). (C) Heatmap showing mean sensitivity to pinprick along the lateral hind paw. Note the broad loss of sensitivity at day 6 in IL-2-complex-treated mice. (D) Area under the curve measurements in IL-2-complex-treated and IgG control mice. Days 1–4: p = 0.9513, t *=* 0.06181; days 5–10: ^∗∗∗^p = 0.0083, t = 2.916; days 11–15: p = 0.4354, t = 0.7951. Student’s unpaired t test was used. (E) Effect of IL-2 complex treatment on NKp46-YFP^+^ cell infiltration to sciatic nerve 6 days after partial crush injury. Sciatic nerve sections (14 μm) were immunolabeled with anti-GFP and β-tubulin III antibodies. Scale bars, 100 μm. Arrows indicate individual YFP^+^ cells. (F) Quantification of NKp46-YFP^+^ cells per square millimeter in images of different regions of the nerve. Two-way ANOVA: effect of IL-2 complex, F(1,16) = 56.31, p < 0.0001; effect of region, F(3,16) = 23.73, p < 0.0001. Bonferroni post-tests: crush, t = 8.062; distal, t = 6.414 ^∗∗∗^p < 0.001. *ns*, not significant. n = 3 sections per region, per mouse, per treatment. (G) Photograph of spleens isolated from mice 1 day after final injection of IgG or IL-2 complex. (H) Peripheral blood sampled 16 days post-injury in IL-2-complex-treated wild-type mice, which received either anti-NK1.1 antibody or isotype control. NKp46^+^DX5^+^ NK cells (t = 15.37, ^∗∗∗^p < 0.0001), CD3^+^CD8^+^ T cells (U = 22.00, p = 0.1473), and CD3^+^CD4^+^ T cells (t = 3.035, p = 0.0079). Student’s unpaired t test was used. (I) Loss of NKp46-YFP^+^ cells from peripheral blood in anti-NK1.1-antibody-treated mice. Student’s unpaired t test, t = 15.51, ^∗∗∗^p = 0.0001. (J) Wild-type mice received either anti-NK1.1 to deplete NK cells or isotype control antibody followed by partial crush of the sciatic nerve on day 0. All mice were treated with IL-2 antibody complex (arrows). Two-way ANOVA. Effect of antibody depletion: F(1,240) = 21.21, p < 0.0001. Bonferroni post-test, ^∗∗∗^p < 0.001 (t = 4.542). (K) Heatmap showing mean sensitivity to pinprick along the lateral hind paw. Note the broad loss of sensitivity at day 6 in isotype control mice. (L) Area under the curve measurements in isotype control and anti-NK1.1-treated mice following IL-2 complex treatment. days 1–4: t *=* 0.1508, p = 0.8815; days 5–10: t = 2.390, p = 0.0295; days 11–15: t = 1.040, p = 0.3130). Student’s unpaired t test was used. See also Figure S5.

Video S5. Live Two-Photon Imaging of NKp46-YFP^+^ Cell “Rolling” along Blood Vessel within Injured Sciatic Nerve *In Vivo*, Related to Figure 5

Video S6. Live Two-Photon Imaging of Highly Motile NKp46-YFP^+^ Cells within in Sciatic Nerve *In Vivo*, Related to Figure 5

### Endogenous NK Response Attenuates Post-injury Sensitivity

Depletion of NK cells by DTx treatment in NKp46-DTR mice (Figure S5E) reduced NKp46^+^ cell infiltration (Figures S5F and S5G) and granzyme B content in the crushed nerves (Figure S5H). Control (PBS-treated) NKp46-DTR mice were almost completely insensitive to pinprick stimulation of the hind paw until a relatively rapid heel-to-toe pattern of recovery beginning in the second week after injury (Figures S5I and S5J, *PBS*) consistent with axon regeneration through the foot (Latremoliere et al., 2018). In contrast, NK depleted mice showed an early and persistent sensitivity to pinprick stimulation (Figure S5I, *DTx*) and displayed an anatomically broad pattern of responses to stimulation across the hind paw prior to full recovery (Figure S5J, *DTx*), indicative of sporadic intact innervation throughout the paw.

### Stimulated NK Cells Degenerate Partially Injured Sensory Axons

To replicate the stimulation of NK cells by IL-2 *in vitro*, we treated mice systemically with an IL-2/anti-IL-2 monoclonal (S4B6) antibody complex (Votavova et al., 2014), leading to enrichment of NKp46^+^DX5^+^ and CD3^+^CD8^+^ lymphocyte populations (Boyman et al., 2006) (Figure 5A). To determine whether NK cells could selectively degenerate partially injured axons, we delivered a reproducible partial sciatic nerve crush (see STAR Methods). Control animals displayed residual pinprick sensitivity 1 day after crush, followed by a rapid functional recovery to over 60% of maximum by day 6 (Figure 5B, immunoglobulin G [IgG]) and reached full recovery by day 15 (Figure 5C, *IgG*), suggesting that a proportion of fibers were not fully axotomized by the initial crush injury. In contrast, animals treated with the IL-2 complex showed a significant acute reduction in the pinprick response 6 days after crush and 1 day after the final injection (Figures 5B, 5C, and 5D, IL-2 complex). This was paralleled by a 10-fold increase in the numbers of NKp46-YFP^+^ cells in the injured nerve at the crush site and distal to the injury, but not proximal to the crush site in IL-2-complex-treated mice (Figures 5E and 5F). Treated mice also displayed enlargement of the spleen (Figure 5G). Contralateral nerves did not display any infiltration of NKp46-YFP^+^ cells (Figures 5E and 5F) and contralateral paws remained fully sensitive to pinprick throughout the experiment (data not shown).

Flow analysis of peripheral blood after recovery (day 16) confirmed depletion of NKp46^+^DX5^+^ NK cells by an anti-NK1.1 antibody in wild-type mice (Figure 5H) and by DTx in NKp46-DTR but not wild-type mice (Figure S5K). No difference was observed in CD3^+^CD8^+^ T cell populations with either method of depletion (Figures 5H and S5L). Anti-NK1.1 antibody was also sufficient to deplete the majority of NKp46-YFP^+^ cells from the peripheral blood of NKp46-YFP mice (Figure 5I). In isotype-control-treated mice, IL-2 complex treatment again resulted in acute loss of the pinprick response (Figures 5J–5L, Isotype), whereas NK cell-depleted mice maintained a constant level of “retained” sensitivity throughout the paw (Figures 5J–5L, Anti-NK1.1). Similar results were obtained in DTx-treated wild-type and NKp46-DTR mice (Figures S5M and S5N).

**Figure S6:**
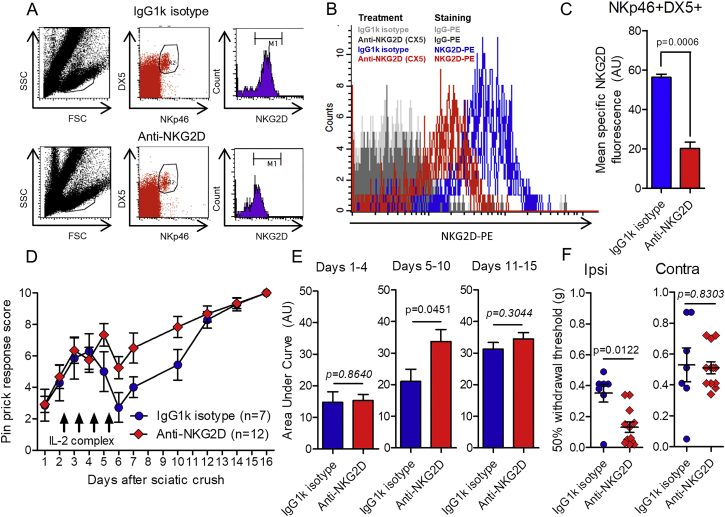
NKG2D Antibody Block Attenuates Effect of IL-2 Complex Treatment on Sensory Recovery after Partial Sciatic Nerve Crush, Related to Figure 6 (A) Adult male C57BL/6 mice received a total of 200 μg anti-mouse NKG2D (CX5 clone) (n = 3) or IgG1k isotype control (n = 3) via retroorbital injection (i.v.) over 4 days (Ogasawara et al., 2004): 50 μg (Day 0), 50 μg (Day 2), 100 μg (Day 4). One day later (Day 5) PBMCs were isolated and labeled with PE-conjugated anti-mouse NKG2D or isotype controls, as well as cell surface NK cell markers NKp46 and DX5. Flow cytometry data were gated on NKp46+DX5+ lymphocytes from 70,000 events. (B) Histogram of NKG2D-PE fluorescence labeling on NKP46+DX5+ gated lymphocytes relative to isotype-PE controls (gray filled). Blue, IgG1k isotype control injection; Red, NKG2D blocking antibody injection. (C) Quantification of mean NKG2D immunofluorescence (IgG isotype subtracted) in NKp46+DX5+ cell population. Student’s t test, t = 9.763, p = 0.0006; n = 3 mice per treatment. (D) Daily pinprick responses in wild-type mice which received either blocking anti-NKG2D antibody (n = 12) or IgGk1 isotype control (n = 7) on days 0, 2 and 4 following partial (moderate) crush of the sciatic nerve. All mice were treated with IL-2/anti-IL-2 antibody complex i.p. daily for four consecutive days from the evening of day 2 (arrows). Two-way ANOVA. Effect of antibody: F(1,187) = 8.03, ^∗∗^p = 0.0051). (E) Area under the curve measurements. Days 1-4: t = 0.1739, p = 0.8640; Days 5-10: t = 2.13, p = 0.0451; Days 11-15: t = 1.059, p = 0.3044. Student’s unpaired t test. (F) Mechanical sensitivity thresholds of ipsilateral (injured: U = 12.00 p = 0.0122, Mann-Whitney U test) and contralateral (uninjured: U = 39.00, p = 0.8303, Mann-Whitney U test) hind paw 16 days after partial crush injury.

IL-2 complex-stimulated wild-type mice treated with the anti-NKG2D blocking antibody, which did not deplete NK cells (Figures S6A), reduced specific NKG2D labeling on peripheral blood NK cells by >60% (Figures S6B and S6C), and attenuated the sensory loss after IL-2 complex treatment compared to isotype-control-treated mice (Figures S6D and S6E).

**Figure 6:**
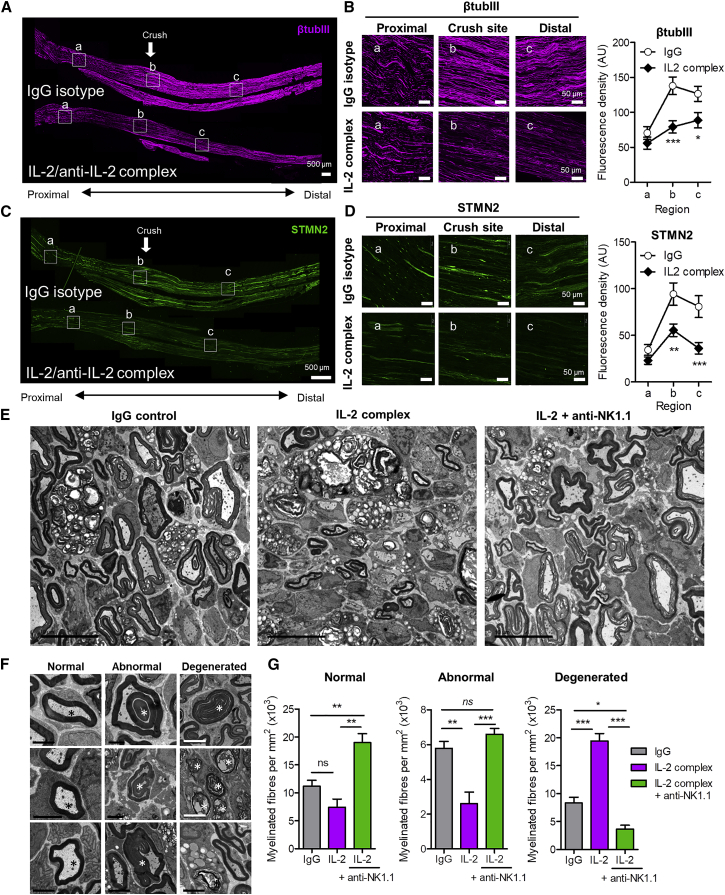
IL-2/Anti-IL2 Antibody Complex after Partial Crush Results in Loss of Myelinated Fibers in Sciatic Nerve (A) β-tubulin III immunolabeling of full-length ipsilateral sciatic nerve sections (14 μm) 6 days after partial crush in mice treated with IgG (top) and IL-2 complex (bottom). Insets a, b, c represent proximal, crush site, and distal regions, respectively. (B) High-magnification images of β-tubulin III immunofluorescence from insets in (A). Scale bars, 50 μm. (Right) Quantification of β-tubulin III fluorescence density of 9,000 μm^2^ area of proximal, crush site, and proximal regions. Two-way ANOVA: effect of region: F(2,64) = 57.39 (p < 0.0001). Effect of IL-2 complex treatment: F(1,64) = 7.36 (p = 0.0106). Bonferroni post-test ^∗^p < 0.05 (t = 2.558), ^∗∗∗^p < 0.001 (t = 3.981). (n = 5 mice per treatment, 3–4 sections per mouse per region, two experimental repeats). (C) Stathmin 2 immunolabeling of full-length ipsilateral sciatic nerve sections 6 days after partial crush in mice treated with IgG (top) and IL-2 complex (bottom). (D) High-magnification images of STMN2 immunofluorescence from insets in (B). Scale bars, 50 μm. (Right) Quantification of STMN2 fluorescence density. Two-way ANOVA: effect of region: F(2,58) = 39.95 (p < 0.0001). Effect of IL-2 complex treatment: F(1,58) = 10.24 (^∗∗^p = 0.0033). Bonferroni post-test ^∗∗^p < 0.01 (t = 3.346), ^∗∗∗^p < 0.001 (t = 3.846). (n = 5 mice per treatment, 3–4 sections per mouse per region. (E) Transmission electron micrographs of cross-sections of ipsilateral sciatic nerve in IgG, IL-2 complex, and anti-NK1.1 antibody treated mice 6 days after partial crush injury. Scale bars, 10 μm. (F) Higher-magnification TEM images showing examples of “normally” myelinated, “abnormally” myelinated, and degenerated axons (denoted by asterisks). Scale bars, 2 μm (black) and 5 μm (white). (G) Quantification of normal myelinated, abnormal myelinated, and degenerated fiber classifications in the different treatment groups. Kruskal-Wallis one-way ANOVA with Dunn’s multiple comparison test. Kruskal-Wallis statistic: normal (26.50), abnormal (25.03), and degenerated (51.81),^∗^p < 0.05, ^∗^p < 0.01, ^∗∗∗^p < 0.001. n = 10 fields of view per nerve section, n = 3 mice per treatment. See also Figure S6.

The density of β-tubulin III-labeled axon fibers in the sciatic nerve 6 days after partial crush was reduced within the crush site and distal region by IL-2 complex treatment (Figures 6A and 6B, insets b and c) but not the proximal nerve region (Figures 6A and 6B, inset a). Labeling for microtubule-associated protein stathmin 2 (STMN2), expressed by injured DRG neurons (Cho et al., 2013), was observed throughout the crushed nerve and was also reduced by IL-2 complex treatment at the crush site and distal regions relative to IgG control (Figures 6C and 6D, regions b and c). STMN2 also co-localized with RAE1 immunolabeling at the injury site (Figures S4B).

To assess axon integrity directly, we employed transmission electron microscopy of the sciatic nerve injury site 6 days after partial sciatic nerve crush (Figure 6E). Myelinated axons, likely to conduct the sensory response to pinprick (Ziegler et al., 1999), were categorized according to their myelin and axoplasm integrity (Figure 6F). Injured nerves from IL-2-complex-treated mice contained significantly more degenerated myelinated axons than IgG-only controls (Figures 6E and 6G, degenerated) and less abnormal axon-Schwann cell units (Figure 6G, abnormal), suggesting that such axon fibers—a feature of clinically relevant peripheral neuropathies (Sander et al., 2000)—may be targeted for degeneration by activated NK cells. Depletion of NK cells with an anti-NK1.1 antibody prior to IL-2 complex stimulation prevented these changes (Figure 6G).

### Clearance of Partially Damaged Axons Alleviates Pain Hypersensitivity after Injury

**Figure 7:**
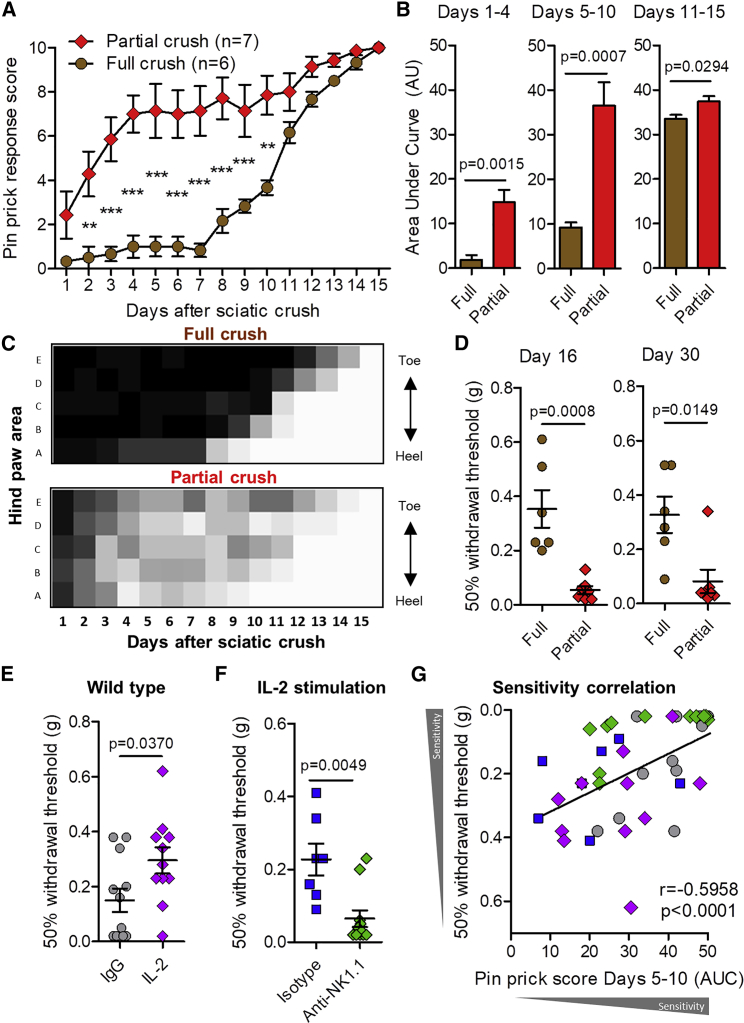
NK Cell Stimulation Post-injury Rescues Long-Term Mechanical Hypersensitivity after Partial Nerve Crush (A) Effect of partial or full crush of the sciatic nerve on daily pinprick responses. Wild-type mice received either partial crush or full crush on day 0. Two-way ANOVA. Effect of crush: F(1,165) = 186.25, p < 0.0001. Bonferroni post-test ^∗∗^p < 0.01 (t = 3.68, 4.07), ^∗∗∗^p < 0.001 (t = 4.19–6.14). (B) Area under the curve measurements showing cumulative difference in pinprick sensitivity between partial and fully crushed sciatic nerve. Student’s unpaired t test: days 1–4, t = 4.176, p = 0.0.0015; days 5–10, t = 4.674, p = 0.0007; days 11–15, t = 2.502, p = 0.0294. (C) Heatmap showing mean sensitivity to pinprick along the lateral hind paw. (D) Mechanical sensitivity thresholds of the ipsilateral hind paw after partial or full crush injury at day 16 (t = 4.550 p = 0.0008, unpaired t test) and 30 days (U = 3.50, p = 0.0149, unpaired Mann-Whitney test) after injury. (E) Mechanical sensitivity thresholds of ipsilateral hind paw 16 days after partial crush injury in mice treated with IL-2 complex or IgG control (U = 32.00, p = 0.0370, Mann-Whitney test). (F) Mechanical sensitivity thresholds of ipsilateral hind paw 15 days after partial crush injury and IL-2/anti-IL-2 complex treatment in mice that received anti-NK1.1 antibody or isotype control (U = 7.000, p = 0.0049, Mann-Whitney test). (G) Mechanical sensitivity outcomes in the injured limb correlated with the cumulative pinprick sensitivity (area under curve, days 5–10) during the peak effect of treatment (Spearman’s correlation: r = −0.5958, p < 0.0001). See also Figure S7.

We next compared the effects of partial and full crush models in the absence of immune modulation (Figures 7A–7C). After recovery of pinprick sensation, paw withdrawal thresholds were significantly lower (i.e., more mechanically sensitive) in mice with previous partial crush injury relative to those that received a full crush, a difference that lasted at least 30 days after the original injury (Figure 7D). IL-2 complex treatment after partial crush resulted in higher hind-paw mechanical thresholds compared to control (IgG) animals at days 15–16 after injury (Figure 7E), an effect that was prevented by either prior depletion of NK cells (Figure 7F) or blockade of the NKG2D-RAE1 interaction (Figure S6F). Combined data revealed a positive correlation between post-crush pinprick sensitivity and the resulting mechanical sensitivity of the injured limb (Figure 7G).

**Figure S7:**
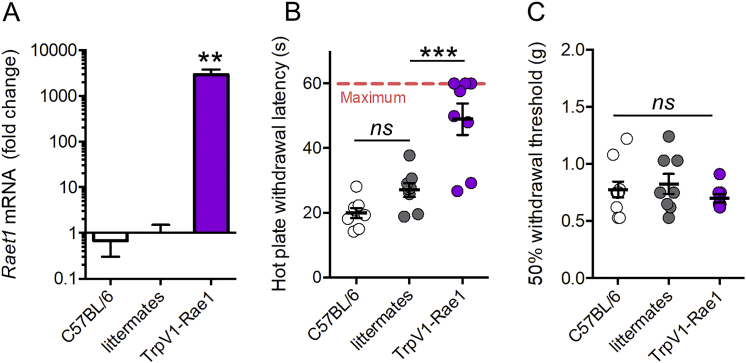
Specific Overexpression of *Raet1* in Nociceptive (TrpV1-Expressing) Neurons Reduces Sensitivity to Noxious Heat but Not Touch, Related to Figure 7 (A) qPCR shows increase in *Raet1* mRNA expression in DRG from TrpV1-Rae1 mice. One-way ANOVA; F (2,18) = 9.346; p = 0.0016 with Bonferroni post-test, t = 3.885, ^∗∗^p < 0.01. n = 5 (C57BL/6), 8 (littermates and TrpV1-Rae1 mice). (B) Latency to withdrawal from a 49°C hotplate. Cut-off time was set at 60 s. TrpV1-Rae1 mice showed reduction on heat pain perception compare to littermate controls. One-way ANOVA; F (2,21) = 22.31; p < 0.0001 with Bonferroni post-test, t = 4.831, ^∗∗∗^p < 0.001). n = 8 mice per genotype. (C) TrpV1-Rae1 mice did not differ from littermate or wild-type controls in their mechanical sensitivity, as measured by the 50% threshold with von Frey filaments of different forces applied to the plantar surface of the hind paw. One-way ANOVA; n = 8 mice per genotype (*ns*, not significant).

Finally, we examined the effect of expression of the RAE1 ligand in sensory neurons in the absence of injury. We used a *Trpv1*-driven Cre mouse line to conditionally overexpress *Raet1* in a population of thermal nociceptive DRG neurons (Figure S7A). These mice displayed an impaired ability to sense noxious heat but demonstrated no change in mechanical thresholds compared to littermates and wild-type controls (Figures S7B and S7C).

## Discussion

### RAE1 Function and Regulation in the Injured Nerve

NKG2D ligands such as RAE1 can be expressed by proliferating tumor cells or at times of cell stress such as during DNA damage or tissue injury (Raulet et al., 2013). Although peripheral nerve injury increased *Raet1* transcription in the DRG, i.e., in cell bodies of sensory neurons, changes in RAE1 protein were detected specifically within the injured sciatic nerve. This suggests that RAE1 protein is either transported anterogradely along the axon or that *Raet1* mRNA is translated locally in injured sensory axons (Willis et al., 2005). The anatomical restriction of RAE1 protein to the peripheral axons of injured sensory neurons suggests that the functional interaction between NKG2D and neuronal RAE1 occurs at the peripheral injury site, which likely protects the cell body from potentially catastrophic attack from NK cells soon after injury (Tandrup et al., 2000). Neurons may also restrict degeneration to the axon by actively inhibiting apoptosis in the cell body (Cusack et al., 2013).

### Recruitment and Cytotoxicity of Injury-Responsive NK Cells

Using two photon imaging *in vivo*, we observed YFP-expressing NK cells rolling and attaching to the blood vessel wall within the injured nerve, suggesting that NK cells enter the nerve via extravasation from the blood, a process known to be chemokine dependent (Sheikh et al., 2004). Consistent with that possibility, the chemokines CCL2, CCL3, and CX3CL1 are all highly expressed after peripheral nerve injury (Ren and Dubner, 2010) with the corresponding chemokine receptors, CCR2, CCR5, and CX3CR1 expressed by NK cells (Loux et al., 2010). Within the nerve tissue itself, YFP-expressing NK cells displayed a “stop-go” behavior, previously observed by live imaging in other tissues (Garrod et al., 2007), allowing NK cells to effectively survey the damaged nerve for injured axons.

In injured sciatic nerve, we detected elevated levels of granzyme B, a serine protease that is essential for NK cell cytotoxic functions (Voskoboinik et al., 2015). Systemic depletion of NK cells reduced levels of granzyme B detected 7 days after nerve injury, suggesting that NK cells are functional in the injured peripheral nerve. *In vitro*, NK cell contact was followed within minutes by intra-axonal Ca^2+^ flux and subsequent neurite fragmentation. This time course is well within the time frame of immune synapse formation between NK and target cells (Lopez et al., 2013) and suggests that targeted delivery of cytotoxic enzymes to the injured axon is sufficient to trigger neurite degeneration (Wang et al., 2012). Further studies will be required to determine whether these NK cell-derived degenerative signals cooperate with the known components of “intrinsic” axon degeneration distal to the injury site, including SARM1 and calpain activation (Gerdts et al., 2016).

### NK Cell-Mediated Neurodegeneration

Wallerian degeneration in the sciatic nerve can be induced by multiple types of crush injury (Bridge et al., 1994). In our study, a conventional forceps crush injury was sufficient to cause the total loss of pinprick sensation from 1 day after nerve injury in all mice, with recovery of evoked sensation beginning more than 1 week later (Latremoliere et al., 2018). However, in NK cell-depleted mice a significant restoration of pinprick response was observed just days following complete crush. This latent sensitivity is in keeping with early electron microscopic studies of Wallerian degeneration, which report that some fibers may survive more than 2 or 3 days and well beyond an early period of axonal degeneration (Dyck and Hopkins, 1972). Our results therefore suggest that certain sciatic nerve crush injuries may not be sufficient to axotomize all fibers and that degeneration of the surviving axons requires an additional extrinsic trigger.

The rapid return of pinprick sensation we observed in the first week after partial crush in control animals is consistent with the functional recovery present in the “moderate” injury model of optic nerve crush (Duvdevani et al., 1990). The increase in β-tubulin III-labeling we observed distal to the partial crush site is further consistent with either sprouting or axon swelling typical after a contusion injury, whereby axons that do not fragment can survive intact long term (Williams et al., 2014). Our electron microscopy data suggest that many axons do indeed survive in the sciatic nerve after partial crush.

IL-2/anti-IL-2 monoclonal (S4B6) antibody complex treatment has been successfully used to expand NK cells *in vivo* for the treatment of cancer (Votavova et al., 2014). This strategy revealed an acute period of NK cell-dependent sensory loss, which correlated with a lower density of STMN2-expressing axons as well as a reduction in abnormally myelinated axons and a corresponding increase in the number of degenerated myelinated axons in sciatic nerve cross-sections. These results suggest that IL-2-activated NK cells further remove damaged axons that would otherwise have been spared. Critically, the simultaneous recovery of pinprick sensation in both groups of mice (by day 15) suggests that successfully regenerating axon fibers are not among those targeted by NK cells.

### Consequences for Post-injury Sensation

After recovery of pinprick responses, partially nerve crushed mice showed hypersensitivity to mechanical stimulation, a surrogate marker of chronic neuropathic pain. The mechanical hypersensitivity that appears 2 weeks after injury may be due to damaged but functionally intact sensory axons, as well as other damaged axons that have not re-innervated their targets but remain within the nerve (Xie et al., 2017). We therefore hypothesize that the active targeting of NK cells to remove partially injured fibers early after injury helps to leave the nerve uncontaminated by previously damaged axons and thus enable successful regeneration without the aberrant sensation and pain often associated with nerve injury (Costigan et al., 2009).

Overexpression of *Raet1* in TRPV1-lineage sensory neurons (Mishra et al., 2011) in otherwise naive mice was sufficient for a loss of sensation specific to noxious heat. This points to the potential for neuronal stressors initiating a detrimental action of NK cells on previously uninjured peripheral nerves (Hickey et al., 1992) by upregulation of NK cell ligands (Backström et al., 2007, Greene et al., 2016). NK cells may therefore be a therapeutic target in multiple die-back peripheral neuropathies that are often co-morbid with metabolic stress in patients with neuropathic pain (Bennett et al., 2014).

## STAR★Methods

### Key Resources Table

**Table:** 

REAGENT or RESOURCE	SOURCE	IDENTIFIER
**Antibodies**		

Rat monoclonal anti-mouse IL-2 (clone S4B6-1)	BioXCell	Cat#BE0043-1; RRID:AB_1107705
Rat IgG2a isotype control (clone 2A3)	BioXCell	Cat#BE0089; RRID:AB_1107769
Rat monoclonal anti-mouse NK1.1 (clone PK136) LEAF purified	Biolegend	Cat#108712; RRID:AB_313399
Rat IgG2a,κ isotype control (clone MOPC-173) LEAF purified	Biolegend	Cat#400224; RRID:AB_326472
Rat monoclonal anti-mouse NKG2D (clone CX5) LEAF purified	Biolegend	Cat#130204; RRID:AB_1227715
Rat IgG1,κ isotype control (clone RTK2071) LEAF purified	Biolegend	Cat#400414; RRID:AB_326520
Rat monoclonal anti-mouse CD16/CD32 (clone 2.4G2)	BD Biosciences	Cat#553142; RRID:AB_394657
Rat monoclonal anti-mouse NKp46 (clone 29A1.4) PE-conjugate	eBioscience	Cat#12-3351; RRID:AB_1210743
Rat monoclonal anti-mouse CD49b (clone DX5) APC-conjugate	eBioscience	Cat#17-5971; RRID:AB_469484
Armenian hamster monoclonal anti-mouse CD3e (clone 145-2C11) FITC-conjugate	eBioscience	Cat#11-0031; RRID:AB_464881
Rat monoclonal anti-mouse CD45 (clone 30-F11) APC-conjugate	eBioscience	Cat#17-0451; RRID:AB_469393
Rat monoclonal anti-mouse CD4 (clone GK1.5) PE-conjugate	eBioscience	Cat#12-0041; RRID:AB_465507
Rat monoclonal anti-mouse CD8a (clone 53-6.7) APC-conjugate	eBioscience	Cat#17-0081; RRID:AB_469335
Rat monoclonal anti-mouse granzyme B (clone NGZB) PE-conjugate	eBioscience	Cat#12-4321; RRID:AB_470052
Rat IgG2a isotype control PE-conjugate	eBioscience	Cat#12-4321; RRID:AB_470052
Rat monoclonal anti-mouse CD314 (NKG2D) (CX5 clone)-PE conjugate	Biolegend	Cat#130207; RRID:AB_1227713
Rat IgG1κ isotype control (Clone MOPC-21)	BD Biosciences	Cat#554680; RRID:AB_395506
Goat polyclonal anti-NKp46	R&D Systems	Cat#AF2225; RRID:AB_355192
Chicken anti-GFP	Abcam	Cat#ab13970; RRID:AB_300798
Rabbit anti-β-tubulin III	Sigma-Aldrich	Cat#T2200; RRID:AB_262133
Chicken anti-NeuN	Merck Millipore	Cat#ABN91; RRID:AB_11205760
Rabbit polyclonal anti-STMN2	Novus Biologicals	Cat#NBP1-49461; RRID:AB_10011569
Goat polyclonal anti-mouse pan-RAE1	R&D Systems	Cat#AF1136; RRID:AB_2238016
Rabbit anti-N-cadherin	Millipore	Cat#04-1126; RRID:AB_1977064
Mouse anti-beta-actin	Sigma-Aldrich	Cat#A5441; RRID:AB_476744
Donkey anti-rabbit Alexa Fluor 647-conjugate	Molecular Probes	Cat#A31573; RRID:AB_2536183
Donkey anti-goat Alexa Fluor 488-conjugate	Jackson Immunoresearch	Cat#705-545-003; RRID:AB_2340428
Goat anti-chicken Alexa Fluor 488-conjugate	Molecular Probes	Cat#A-11039; RRID:AB_142924
Goat anti-chicken Biotin-conjugate	Vector	Cat#BA-9010; RRID:AB_2336114
Donkey anti-goat HRP-conjugate	Santa Cruz	Cat#sc-2020; RRID:AB_631728
Goat polyclonal anti-mouse HRP-conjugate	Komabiotech	Cat#K-0211589; RRID:AB_2636911
Goat anti-rabbit HRP-conjugate	Santa Cruz	Cat#sc-2004; RRID:AB_631746

**Chemicals, Peptides, and Recombinant Proteins**		

Recombinant mouse interleukin-2	Peprotech	Cat#212-12, Lot#0608108
Collagenase A	Roche	Cat#10-103-578-001
Dispase II	Roche	Cat#09-942-078-001
DNase I	Sigma-Aldrich	Cat#D5025
Trypsin	GIBCO, Life Technologies	Cat#15090-046
Trypsin inhibitor	Sigma-Aldrich	Cat#T9003
Nerve growth factor (NGF) 2.5S	GIBCO, Life Technologies	Cat#13257-019
Poly-D-lysine	Sigma-Aldrich	Cat#P6407
Laminin	Sigma-Aldrich	Cat#L2020
Penicillin-Streptomycin	GIBCO, Life Technologies	Cat#15140-122
Vybrant DiI	Molecular Probes	Cat#V-22886
Rhodamine 3-AM	Molecular Probes	Cat#R10145
Pacific blue streptavidin	Thermo Scientific	Cat#S11222
Normal donkey serum	ImmunoResearch	Cat#017-000-121

**Critical Commercial Assays**		

Mouse NK cell isolation Kit II	Miltenyi Biotech GmbH	Cat#130-096-892
Pierce LDH Cytotoxicity Assay Kit	Thermo Scientific	Cat#88954
BD Cytofix/Cytoperm	BD Biosciences	Cat#554714
Granzyme B ELISA DuoSet	R&D Systems	Cat#DY1865
RNeasy Plus	QIAGEN	Cat#74134
RNA Scope 2.5 HD assay (Red)	Advanced Cell Diagnostics	Cat#322350
Mm-Raet1 probe	Advanced Cell Diagnostics	Cat#448121;

**Experimental Models: Organisms/Strains**		

Mouse: B6.129X1-Gt(ROSA)26Sor < tm1(EYFP)Cos > /J	The Jackson Laboratory	RRID: IMSR_JAX:006148
Mouse: C57BL/6-Gt(ROSA)26Sor < tm1(HBEGF)Awai > /J	The Jackson Laboratory	RRID: IMSR_JAX:007900
Mouse: B6(Cg)-Ncr1 < tm1.1(icre)Viv > /Orl	Narni-Mancinelli et al., 2011	RRID: MGI:5308422
Mouse: B6.129-Trpv1tm1(cre)Bbm	The Jackson Laboratory	RRID: IMSR_JAX:017769
Mouse: C57BL/6-Gt(ROSA)26Sor < tm1(Raet1e)Lll	Morvan et al., 2017	RRID: MGI:5823988

**Oligonucleotides**		

siRNA targeting sequence Raet1a-e: Sense: CAAUGGUUACCCACAUUUAtt Anti-sense: UAAAUGUGGGUAACCAUUGgt	Silencer Select, Ambion, Life Technologies	Cat#4390771; ID s234333
Negative control siRNA #1	Silencer Select, Ambion, Life Technologies	Cat#4390843
See Table S1 for primer sequences.	This paper	N/A

**Software and Algorithms**		

ImageJ v1.46r	NIH, USA	https://imagej.nih.gov/ij
ZEN 2012 v8.1 SP1	Zeiss	https://www.zeiss.com/microscopy/int/downloads.html?vaURL=www.zeiss.com/microscopy/int/downloads/zen.html#service-packs
ElisaAnalysis	Elisakit.com Pty	https://elisaanalysis.com/
GraphPad Prism version 5.00 for Windows	GraphPad Software, San Diego California USA	https://www.graphpad.com/

### Contact for Reagent and Resource Sharing

Further information and requests for resources and reagents should be directed to and will be fulfilled by the Lead Contact, Seog Bae Oh (odolbae@snu.ac.kr).

### Experimental Model and Subject Details

#### Animals

All procedures were approved by the Institutional Animal Care and Use Committee (IACUC) at Seoul National University (Approval number: SNU-121011-1). Adult male and pregnant dam wild-type C57BL/6 mice were purchased from Dae Han Bio Link (Taconic, Korea). *Rosa26*^*eyfp*^ (RRID:IMSR_JAX:006148), *Rosa26*^*dtr*^ (RRID:IMSR_JAX:007900) and *Trpv1*^*cre*^ mice which express Cre recombinase from the endogenous *Trpv1* locus (RRID:IMSR_JAX:017769), were purchased from Jackson Laboratories (USA). *Ncr1*^*icre*^ mice (RRID:MGI:5308422), containing cre-recombinase inserted by homologous recombination at the 3′ end of the *Ncr1* (*Nkp46*) gene (Narni-Mancinelli et al., 2011), were a kind gift from Dr Eric Vivier. *Rosa26*^*raet1e*^ mice (RRID: MGI:5823988), expressing a floxed stop site upstream of the *Raet1e* gene inserted at the Rosa26 locus (Morvan et al., 2017) were a kind gift from Dr Lewis Lanier. All mice are originally on a C57BL/6 background except *Trpv1*^*cre*^, which are on a mixed B6 and 129 background, and *Rosa26*^*eyfp*^ mice which were backcrossed onto the C57BL/6 background for at least 10 generations. All mice were maintained as homozygous stocks in a specific pathogen free (SPF) facility and transferred to a conventional room at least one week before experiments. Double heterozygote *Nrc1*^*icre/wt*^*;rosa26*^*eyfp/wt*^ mice (abbreviated to NKp46-YFP) and *Nrc1*^*icre/wt*^*;rosa26*^*dtr/wt*^ mice (abbreviated to NKp46-DTR) were bred from single crosses. *Trpv1*^*cre/wt*^*;rosa26*^*raet1e/wt*^ mice (abbreviated to TrpV1-Rae1) and *Trpv1*^*cre/wt*^*;rosa26*^*wt/wt*^ mice (littermates) were the product of crosses between *Trpv1*^*cre/cre*^ and *rosa26*^*raet1e/wt*^ mating pairs. Mice were maintained on a 12 h:12 h light/dark cycle (lights on at 8:00 a.m.), housed 4-6 mice per cage on wood chip bedding with nestlets and provided with standard laboratory feed and water *ad libitum*. DRG neurons and NK cells were prepared from male C57BL/6 mice (6-8 weeks); embryonic DRG neurons were prepared from embryos on day 15 in utero (E15) removed from euthanized female C57BL/6 mice. Nerve injury experiments were performed on male mice age 7-9 weeks old of indicated genotype. Animals were killed in accordance with Schedule 1 of the UK Animals (Scientific Procedures) Act 1986 by inhalation of a rising lethal concentration of isoflurane followed by decapitation for tissue culture, or a rising lethal concentration of carbon dioxide at the end of experiments.

### Method Details

#### NK cell depletion

NKp46-DTR mice were treated with diphtheria toxin (DTx; 100 ng) or sterile PBS vehicle (100 μl) intravenously by retro-orbital injection. Under isoflurane anesthesia (3% induction, 1%–2% maintenance in 100% O_2_) an ophthalmic solution (0.5% proparacaine, Alcon, Belgium) was applied to the eye as a local anesthetic. An insulin syringe (0.3ml, BD Biosciences) was inserted bevel-down behind the eye into the retro-orbital sinus and 100 μl slowly injected. Injections were alternated between eyes. Injections were administered blind to the content of the syringe starting one day prior to surgery and continuing every four to five days for the duration of the study. All mice were blood sampled at the end of experiments for depletion efficiency check by flow cytometry.

For antibody depletion of NK cells, wild-type C57BL/6 mice were injected with 100 μg of LEAF purified anti-mouse NK1.1 (clone PK136) (Biolegend, cat no. 108712, RRID:AB_313399) or LEAF purified rat IgG2a,κ isotype control (clone MOPC-173) (Biolegend, cat no. 400224, RRID:AB_326472) intravenously by retro-orbital injection one day before nerve injury.

For blocking of NKG2D *in vivo* adult male C57BL/6 mice received a total of 200 μg LEAF purified anti-mouse CD314 (NKG2D) (CX5 clone) (Biolegend, cat no. 130204, RRID:AB_1227715) or LEAF purified rat IgG1k isotype control (Clone RTK2071) (BioLegend, cat no. 400414, RRID:AB_326520) via retroorbital injection (i.v.) over 4 days: 50 ug (Day 0), 50 ug (Day 2), 100 ug (Day 4).

#### L5 spinal nerve transection (L5x)

Male mice age 7-9 weeks old were placed under isoflurane inhalation, the dorsal lumbar region was shaved, treated with an iodine solution (Potadine) and a unilateral incision made parallel to the L6 vertebrate. Under a x20 dissection microscope illuminated by a cold light source the musculature was parted by blunt forceps dissection to reveal the L6 transverse process, which was then cut and removed. The L5 spinal nerve, which runs immediately below the L6 process, was carefully freed of connective tissue and cut with fine spring scissors; 1 mm of the nerve was removed to prevent nerve regeneration. The wound was irrigated with sterile saline and closed in two layers with 6-0 silk sutures (Ailee, Korea) and 9mm skin clips (MikRon Precision, CA, USA) Mice were placed in a warm, darkened cage to recover from surgery.

#### Sciatic nerve crush

Adult male mice (7-9 weeks old) received a single unilateral crush injury to the sciatic nerve (Bridge et al., 1994). Briefly, under isoflurane anesthesia the right thigh was shaved and iodine treated and an incision was made mid-thing length. The sciatic nerve exposed as it emerges from the sciatic foramen by parting the muscle with blunt forceps dissection. The nerve was carefully freed of connective tissue and fully crushed for 15 s using fine, mirror-finished forceps (No 5, Dumont, Fine Science Tools, Germany). The wound was closed in two layers with two sutures of the overlying muscle facia and a single skin clip to close. Complete crush of the sciatic nerve was deemed successful by a sensory score of zero using the pinprick assay on the day following surgery; any mice with a pinprick response less than 24 h after surgery were excluded from analysis.

For partial crush the sciatic nerve an ultra-fine hemostat (Cat no. 13020-12, Fine Science Tools, Germany) was fitted with a custom spacer created from two layers of aluminum foil (15 μm thick) to create a gap 30 μm thick when fully closed. The sciatic nerve was carefully freed of connective tissue and placed between spacers on the hemostat (2-3 mm from the tip) by gently lifting the nerve using a fire-polished glassed rod. The hemostat was then closed on the first locking position and held for 15 s before careful release of the nerve. Full sciatic nerve crush was performed in the same way except no spacer was used. The wound was closed in two layers with two sutures of the overlying muscle facia and a single skin clip. Mice were recovered in a warm, darkened cage. All tools were autoclaved prior to surgery and strict aseptic technique was maintained throughout.

#### IL-2/anti-IL-2 antibody complex treatment

Recombinant mouse interleukin-2 (Cat no. 212-12, lot no. 0608108; Peprotech, Rocky Hill, NJ, USA) was prepared as a stock at 0.1 mg/ml in PBS (without carrier protein) and stored at 4°C for up to one week, according to the manufacturer’s instructions. On the day of treatment, IL-2 (1.5 μg per mouse) was pre-mixed with anti-mouse IL-2 monoclonal antibody (50 μg per mouse) (S4B6-1 clone) (BioXCell; RRID:AB_1107705) and incubated at room temperature for 15 min. The bound cytokine/antibody complex was further diluted in sterile PBS to a total volume in 500 μl and injected intraperitoneally. Injections were given once daily (evening time) for four days. For control experiments, mice were injected with an equal amount of rat IgG2a isotype peptide (clone 2A3) (BioXCell; RRID:AB_1107769). The efficacy of the IL-2 antibody complex treatment was confirmed by enlargement of the spleen compared to PBS injected mice one day after the final injection (Boyman et al., 2006).

#### Behavioral testing

All sensory testing was performed between the hours of 09.00 and 18.00 in an isolated room maintained at 22 ± 2°C and 50 ± 10% humidity. For mechanical threshold (von Frey filament) testing, mice were brought from the animal colony and placed in transparent plastic boxes on a metal mesh floor with 5x5 mm holes (Ugo Basile, Italy). The mice were then habituated for at least 30 min prior to testing. To assess mechanical sensitivity, the withdrawal threshold of the affected hind paw was measured using a series of von Frey filaments (0.20, 0.40, 0.70, 1.6, 3.9, 5.9, 9.8 and 13.7 mN, Stoelting, Wood Dale, IL, USA; equivalent in grams to 0.02, 0.04, 0.07, 0.16, 0.40, 0.60, 1.0 and 1.4). The 50% withdrawal threshold was determined using the ‘up-down’ method and calculated using Up-Down Reader software (Gonzalez-Cano et al., 2018). A brisk hind paw lift or flinch in response to von Frey filament stimulation was regarded as a withdrawal response. The 0.4 g filament was the first stimulus to be used, and, when a withdrawal response was obtained, the next weaker filament was used. This process was repeated until no response was obtained, at which time the next stronger filament was administered. All behavioral testing was performed by an investigator who was blind to the treatment of the mice.

Pinprick sensory recovery testing was performed as previously described (Latremoliere et al., 2018) with slight modification. Mice were habituated on an elevated mesh in separate compartments. The lateral side of the affected hind paw was separated into five regions from the toe to the heel and stimulated with a stainless steel Austerlitz insect pin (Size 000, FST, Germany). A sensory response was confirmed by rapid lifting or flinching of the paw. The number of responses to two consecutive pin applications to the skin was recorded per region providing a score out of 10. Responses due to direct movement of the paw or hind limb (indicative of extraterritorial proprioception) were excluded. Testing was carried out daily (09.00 – 14.00) until full sensory recovery (score of 10) was observed for all mice.

For hot plate testing, mice were placed on a metallic plate heated at 49°C within an acrylic container (Bioseb, France), and the latency to flinching, licking one of the hind paws or jumping was measured. A cut-off time of 60 s was used to minimize the possibility of cutaneous tissue damage.

#### DRG culture

Adult and embryonic DRG were rapidly dissected on ice-cold Ca^2+^- and Mg^2+^-free Hank’s Buffer Saline Solution (HBSS, Welgene) (including 20mM HEPES) and digested 30-60 min in collagenase A (1 mg/ml) and dispase II (2.4U/ml) (Roche, Switzerland) at 37°C. Additional digestion was carried out for 5-7 min in trypsin (0.25%) and stopped with a trypsin inhibitor (2.5 mg/ml) (Sigma, T9003) in PBS followed by washing in Dubellco’s Modified Eagle Medium (GIBCO, Life Technologies) containing 10% serum (GIBCO, Life Technologies). DRG were dissociated by trituration with a fire-polished glassed pipette in DMEM containing DNase I (125 U/ml) and centrifuged at 200 g on a layer of Bovine Serum Albumin (15% BSA solution; Sigma) before re-suspension in neurobasal medium (GIBCO, Life Technologies) with B27 supplement, L-glutamine (1 mM), penicillin (100 U/ml) and streptomycin (100 U/ml) supplemented with nerve growth factor (NGF 2.5S) at 50 ng/ml. Adult DRG (10^3^ cells) and embryonic DRG (8x10^3^ cells) were plated on 10 mm diameter glass coverslips, glass bottom dishes or 96 well flat-bottom culture plates (Nunclon, Thermo Scientific) previously coated with poly-D-lysine (10 μg/ml) and laminin (10 μg/ml) (Sigma).

For injured DRG experiments, ipsilateral L5 DRG were rapidly dissected on ice-cold HBSS from adult mice 7 days after L5x or sham surgery, pooled (n = 3 DRG per group) and dissociated to a single cell suspension as above. DRG were then seeded onto poly-D-lysine and laminin-coated glass bottom dishes (10^3^ cells per dish) and cultured overnight in neurobasal medium containing NGF (50 ng/ml).

For microfluidic co-cultures, DRG neurons isolated from adult mice (as above) were suspended in neurobasal medium and seeded (10^4^ cells) into the somal reservoir of a microfluidic device (Xona Microfluidics, CA, USA) previously coated with poly-D-lysine (10 μg/ml) and laminin (10 μg/ml) (Sigma). NGF (100 ng/ml) was added to the media in the neurite reservoir. Neurons were cultured for 5 days during which neurites grew along 3 μm x 500 μm channels connected to the neurite reservoir.

#### NK cell isolation and stimulation *in vitro*

NK cells were prepared from adult male C57BL/5 mouse spleens (6-8 weeks old). Spleens were homogenized by sequentially passing through 70 μm and 40 μm cell strainers (Falcon, BD Biosciences). Red blood cells were lysed by incubation for 2 min in ACK lysis buffer (in mM: 150 NH_4_Cl, 10 KHCO_3_, 0.1 Na_2_EDTA, pH 7.3). Single-cell suspensions were then passed through nylon wool columns (Polysciences, Warrington, PA) for the depletion of adherent populations, consisting of B cells and macrophages. Eluted cells were re-suspended in 0.01 M phosphate buffered saline (PBS) plus 2 mM EDTA and 2% FBS. NK cells were enriched using a magnetic associated cell sorting (MACS) method in combination with a negative selection protocol (Mouse NK cell isolation Kit II, cat no. 130-096-892, Miltenyi Biotech GmbH, Germany) according to the manufacturer’s instructions. Briefly, cell suspensions were sequentially incubated at 4°C with a cocktail of biotin-conjugated monoclonal antibodies against non-NK cells followed by anti-biotin microbeads. The cell suspension was then passed through an LS column placed in a magnetic field (MidiMACS Separator, Miltenyi Biotech GmbH). Bead-conjugated non-NK cells remained in the columns while non-labeled NK cells passed through in the eluent. Enriched NK cell were either used directly (control) or stimulated with recombinant murine interleukin (IL)-2 (Cat no. 212-12, lot no. 0608108; Peprotech, Rocky Hill, NJ, USA) at 1000 U/ml for two days before use in experiments. NK cells were cultured at 2x10^6^ cells per ml in RPMI 1640 medium (GIBCO, Life Technologies) supplemented with fetal bovine serum (FBS) (10%) and penicillin/streptomycin (100 U/ml) in 96-well U-bottomed (Falcon, BD Biosciences) plates for 48 h. The purity of NK cells (NKp46+DX5+) in the eluent was checked by flow cytometry to be consistently above 90%. The cells were then harvested and used as effector cells in co-culture and cytotoxicity experiments.

#### DRG - NK co-cultures

Control or IL-2 stimulated NK cells were harvested, washed in RPMI and re-suspended in neurobasal media. DRG were washed once in neurobasal media (NGF-free) and NK were added to the neurite compartment (5x10^5^ cells) for microfluidic cultures or seeded (2.5x10^5^ cells) directly over the DRG for glass coverslip cultures, and co-cultured for 4 h at 37°C and 5% CO_2_. Co-cultures were carefully washed once in warm HBSS and fixed with 2%–4% PFA in 0.01M PBS (pH 7.4) for 30 min at RT, followed by washing in PBS (3 × 10 mins) at storage at 4°C prior to immunolabeling for β-tubulin III and NKp46 (see *Immunofluorescence*).

For transwell experiments, glass coverslip cultures of DRG were transferred to a 24-well plate in 500 μl Neurobasal media. NK cells (2.5x10^5^ per well) were seeded onto 6.5 mm diameter polycarbonate transwell membrane inserts with 0.4 μm pore size (Corning) and incubated for 4 h at 37°C and 5% CO_2_ before removal of the membrane and fixation as above.

For antibody blockade of NKG2D function *in vitro*, NK cells were incubated with 30 μg/ml of LEAF purified anti-mouse CD314 (NKG2D) (CX5 clone) (Biolegend, cat no. 130204. RRID:AB_1227715) or LEAF purified rat IgG1 isotype control (Clone RTK2071) (Biolegend, cat no. 400414. RRID:AB_326520) for 15 min at room temperature (2.5x10^6^ NK cells per ml) before addition to target DRG neurons.

#### LDH-release cytotoxicity

Effector NK cells were assayed for cytotoxicity against DRG neuron targets by measuring the release of lactate dehydrogenase (LDH) into the culture medium using an LDH Cytotoxicity Assay Kit (Thermo Scientific Pierce, IL, US). Control or IL-2 stimulated NK cells were harvested, washed in RPMI and added to DRG cultures in 96 well plates (Nunclon, Thermo Scientific) in neurobasal medium at various ratios and cultured for 4 h before sampling of media supernatant which was assayed for LDH activity according to manufacturer’s instructions. Absorbance values were acquired on a microplate spectrophotometer (BioTek Instruments, VT, US). Each ratio was determined in triplicate. Specific cytotoxicity was calculated as follows: % cytotoxicity = [(experimental release - spontaneous release)/(maximum release - spontaneous release)] x 100. The optimum number of target cells (DRG neurons) for maximum LDH release detection was determined prior to experiments (8x10^3^ embryonic DRG neurons; 10^3^ adult DRG neurons).

#### Live confocal imaging

Adult or embryonic mouse DRG (10^3^ and 8x10^3^ neurons, respectively) cultured for one day on PDL and laminin-coated glass-bottom Petri dishes were fluorescently labeled with Vybrant DiI (Molecular Probes, cat no. V-22886) or loaded with the Ca^2+^ indicator rhodamine 3-AM (Molecular Probes, cat no. R10145) according to the manufacturer’s instructions. NK cells previously isolated from NKp46-YFP mice and stimulated IL-2 (1000U/ml) for 48 h were suspended in neurobasal media and seeded onto the coverslip (2.5x10^5^ cells per dish). Dishes containing DRG-NK co-cultures were immediately transferred to a confocal microscope (LSM 700, Zeiss) and maintained in a humidified atmosphere at 37°C and 5% CO_2_ (Live Cell Instruments, Seoul Korea). A time series of single z section images (512 × 512) were acquired using a multitrack setting (488 nm and 555 nm fluorescence emission and differential interference contrast (DIC) bright-field) at 30-60 s intervals and multiple positions under the control of Definite Focus. Images were acquired up to 3h and exported as a sequential time-lapse in AVI format.

#### *In vivo* two photon sciatic nerve imaging

Male NKp46-YFP mice (8-9 weeks old) received unilateral sciatic nerve crush or sham surgery. On day 3 mice were anaesthetized with pentobarbital (80 mg/kg, i.p.) supplemented with 20 mg/kg immediately prior to recording. Some mice additionally received Dextran-Texas red (neutral 40,000 m.w.) (Molecular Probes) given via retro-orbital injection (100 μl, 10 mg/ml) to visualize the vasculature and confirm maintenance of blood flow during recording. The sciatic nerve was re-exposed and bathed in sterile saline. Mice were placed on a warm pad maintained at 35°C for the duration of the imaging. The sciatic nerve was carefully lifted with two curved glass rods held via a micromanipulator at both distal sides of the exposed nerve to mechanically isolate the nerve from the body and minimize the breathing-induced motion artifacts. A W plan-Apochromat 20X water immersion lens was lowered to the nerve surface 1-2 mm distal from the crush site to identify the blood vessels and YFP-positive cells. After the Ti-sapphire laser (Chameleon, COHERENT) was tuned to the wavelength of 900 nm for excitation of both YFP and Texas Red, 3D time-lapse imaging was performed using a laser scanning confocal microscope (LSM 7MP, Zeiss) as follow: 1024 × 512 pixels per a single image, 0.79 μs of pixel dwell, 2 μm x 25 sections per a z stack (total 50 μm depth) at 30 s interval. The two-photon laser power was compensated according to the depth across 5 - 8% of total power. The image was rendered into 2D video using imaging software (Zeiss Efficient Navigation 2012).

#### DRG small interference RNA gene knockdown

Acute isolated adult or E15 embryonic DRG were transfected with siRNA by electroporation (Neon, Invitrogen) according to the manufacturer’s instructions. Briefly, single cell suspensions of DRG were suspended in an electroporation medium containing siRNA oligonucleotide and drawn into a tip containing a gold-plated electrode (5x10^4^ cells per 10μl). Tips of cells were placed into a tube containing electrolytic buffer, electroporated (1500V, 20ms) and immediately ejected into penicillin/streptomycin-free neurobasal media containing 50 ng/ml NGF and cultured for 48h at 37°C, 5% CO_2_ until assessment of assessment of knockdown or functional experiments. Prior to experiments transfection efficiency was optimized by electroporation of DRG with a cDNA plasmid encoding green fluorescent protein (GFP) (0.5μg) and examination of GFP fluorescence after two days in culture. Two siRNA oligonucleotides per target were tested for knockdown efficiency by real time PCR; siRNA oligos that reduced mRNA expression by more than 70% compared to a negative control siRNA oligonucleotide were used for functional experiments. *Gapdh* siRNA oligonucleotides (10nM) (Silencer Select, Ambion, Life Technologies, cat no. 4390849) were used as a positive control. siRNA oligonucleotides used for cytotoxicity assays were: *Raet1a-e* (10nM) (sense) 5′-CAAUGGUUACCCACAUUUAtt-3′; (antisense) 5′-UAAAUGUGGGUAACCAUUGgt-3′ (Silencer Select, Ambion, Life Technologies, cat no. 4390771, ID s234333); Negative control #1 (10nM) (Silencer Select, Ambion, Life Technologies, cat no. 4390843).

#### Peripheral blood lymphocyte isolation

Systemic depletion of NK cell was confirmed at the end of experiments in a sample of peripheral blood obtained by retro-orbital bleed. Briefly, under isoflurane anesthesia a 15ml glass Pasteur pipette was inserted into the retro-orbital sinus and gently twisted to disrupt the orbital venous plexus. 50-100 μl of blood was removed by capillary action and ejected into heparin-coated tubes (Idexx Laboratories, USA). Blood samples were diluted with an equal volume of serum-free RPMI, floated on top of a lymphocyte separation medium (Lympholyte Mammal, Cedarlane Labs, Canada) and centrifuged at 1000 g for 20 min with a slow acceleration gradient. Peripheral blood mononuclear cells were collected from the monolayer, washed in RPMI and suspended in FACS buffer (5% FBS, 0.002% NaN_3_ in 0.01M PBS, pH 7.4) for flow cytometry analysis.

#### Flow cytometry

For surface labeling cell suspensions were transferred to 5 mL round bottom tubes and Fc receptors were blocked with unconjugated rat anti-mouse CD16/CD32 (clone 2.4G2) monoclonal antibody (1:100; BD Biosciences, cat no. 553142. RRID:AB_394657) for 15 min at 4°C and labeled with a combination of fluorescently-conjugated anti-mouse primary antibodies for 30 min at 4°C. After washing with FACS buffer cell suspensions were run on a four-color flow cytometer (FACSCalibur, BD Biosciences) and gated populations analyzed with Cell Quest software (BD Biosciences). Lymphocytes were initially gated according to the FSC-SSC scatter profile; cell populations were identified by fluorescence gating compared to unlabeled or IgG controls. Antibodies used were PE rat anti-mouse NKp46 (clone 29A1.4) monoclonal antibody (1:200; eBioscience, cat no. 12-3351. RRID:AB_1210743), APC rat anti-mouse CD49b (clone DX5) monoclonal antibody (1:500; eBioscience, cat no. 17-5971. RRID:AB_469484), FITC Armenian hamster anti-mouse CD3e (clone 145-2C11) monoclonal antibody (1:1000; eBioscience, cat no. 11-0031. RRID:AB_464881), APC rat anti-mouse CD45 (clone 30-F11) monoclonal antibody (1:200; eBioscience, cat no. 17-0451. RRID:AB_469393), PE rat anti-mouse CD4 (clone GK1.5) monoclonal antibody (1:1000; eBioscience, cat no. 12-0041. RRID:AB_465507), APC rat anti-mouse CD8a (clone 53-6.7) monoclonal antibody (1:1000; eBioscience, cat no. 17-0081. RRID:AB_469335). For confirmation of NKG2D block *in vivo* mean specific fluorescence labeling with PE anti-mouse CD314 (NKG2D) (CX5 clone) (Biolegend, cat no. 130207. RRID:AB_1227713) was calculated by subtracting the mean fluorescence intensity obtained with PE rat IgG1κ isotype control (Clone MOPC-21) (BD Biosciences, cat no. 554680. RRID:AB_395506). Titers of flow cytometry antibodies were determined prior to experiments by comparison to equivalent concentrations of fluorescence-conjugated IgG isotype controls. Percentages of peripheral blood cell populations were calculated from 20,000 gated lymphocyte events.

#### Intracellular staining

For intracellular labeling of granzyme B cell suspensions were washed in RPMI, resuspended in FACS buffer and Fc receptors were blocked for 15 min at 4°C. Cells then underwent fixation and permeablisation in BD Cytofix/Cytoperm buffer (BD Biosciences, cat no. 554714) for 20 min at 4°C followed by labeling with PE rat anti-mouse granzyme B (clone NGZB) monoclonal antibody (1:100; eBioscience, cat no. 12-8898. RRID:AB_10853811) or PE rat IgG2a isotype control (1:100; eBioscience, cat no. 12-4321. RRID:AB_470052) for 30 min at 4°C and washing in BD Perm/Wash buffer.

#### Preparation of sciatic nerve suspensions and flow cytometry

For flow cytometry analysis of NK cells present in sciatic nerve, NKp46-YFP mice were deeply anaesthetized with pentobarbital (100mg/kg, i.p.) followed by trans-cardiac perfusion with PBS (0.01 M, pH 7.4) at various time points after peripheral nerve injury to remove peripheral blood from the circulation. Bilateral sciatic nerves were rapidly removed to ice-cold Ca^2+^- and Mg^2+^-free HBSS (Welgene) (including 20mM HEPES) and cut into 1-2 mm pieces. Tissues were transferred to 15 mL tubes and centrifuged at 500 g for 5 min. HBSS was replaced with collagenase A (1 mg/ml) and dispase II (2.4 U/ml) (Roche, Switzerland) and incubated 90 min at 37°C with frequent gentle agitation. Additional digestion was carried out for 5 min in trypsin (0.25%) and stopped with a trypsin inhibitor (2.5 mg/ml) (Sigma, T9003) in PBS followed by washing in RPMI containing 10% serum (GIBCO, Life Technologies). Nerves were dissociated by trituration with a fire-polished glassed pipette in RPMI containing DNase I (125 U/ml), passed through a 30 μm separation filter to remove debris (Miltenyi) and resuspended in FACS buffer in 5 mL round bottom tubes. Prior to sciatic nerve flow cytometry, CD45+ lymphocyte and NKp46-YFP cell gating was set on peripheral blood lymphocytes from wild-type and NKp46-YFP mice. Sciatic nerve samples were run on the slow setting until the total event count was less than 50 per second, or gated events were less than one per 5 s. NK cells were identified by lymphocyte FSC-SSC scatter profile and YFP fluorescence; in some experiments total lymphocytes were additionally labeled with an APC-conjugated anti-mouse CD45 antibody (1:400; eBioscience, cat no. 17-0451. RRID:AB_469393).

#### Tissue preparation

At specific time points after nerve injury or sham surgery, mice were deeply anaesthetised with pentobarbital (100 mg/kg, i.p.) followed by exsanguination by trans-cardiac perfusion with PBS (0.01 M, pH 7.4) containing heparin (500 U/L). Whole DRG (lumbar L3-L5) and full length sciatic nerve (spinal nerves to peripheral trifurcation) tissues were dissected and collected in sample tubes which were immediately frozen on liquid nitrogen and stored at −70°C for later molecular analysis. For immunohistochemistry PBS perfusion was followed by a paraformaldehyde fixative (4% PFA, 0.2% picric acid in 0.1M PBS, pH 7.4). Sciatic nerves were post-fixed up to 24h and cryoprotected in sucrose solution (30% sucrose in 0.01M PBS, pH 7.4) at 4°C. Fixed sciatic nerve tissues were embedded in frozen section compound (Optimal Cutting Temperature, Leica), sectioned on a cryostat (14μm) and thaw-mounted on glass microscope slides (Superfrost Plus, Fischer Scientific).

#### Immunofluorescence

After washing in PBS (3 × 10 mins) cells and tissues were blocked and permeabilized in 10% normal donkey serum (NDS, Jackson ImmunoResearch) and 0.1%–0.3% Triton X-100 in PBS for 1h at RT. Primary antibodies were applied in 1% NDS, 0.01%–0.03% Triton X-100 in PBS and incubated overnight at 4°C in a humidified chamber. Primary antibodies used were: Rabbit anti-β-tubulin III (1:400-500; Sigma, cat no. T2200. RRID:AB_262133), goat anti-NKp46 (1:200; R&D Systems, cat no. AF2225. RRID:AB_355192), rabbit anti-STMN2 (1:500; Novus Biologicals, cat no. NBP1-49461. RRID:AB_10011569), goat anti-mouse pan-RAE1 (1:40; R&D systems, cat no. AF1136. RRID:AB_2238016), chicken anti-GFP (1:2000; Abcam, cat no. ab13970. RRID:AB_300798) and chicken anti-NeuN (1:400; Merck Millipore, cat no. ABN91. RRID:AB_11205760). Fluorescence-conjugated secondary antibodies were incubated in 1% NDS, 0.1%–0.3% Triton X-100 in PBS for 1h at RT the dark. Secondary antibodies used were: Alexa Fluor 647 donkey anti-rabbit (1:200; Molecular Probes, cat no. A31573. RRID:AB_2536183), Alexa Fluor 488 donkey anti-goat (1:200; Jackson ImmunoResearch, cat no. 705-545-003. RRID:AB_2340428), Alexa Fluor 488 goat anti-chicken (1:200; Molecular Probes, cat no. A-11039. RRID:AB_142924), Biotin goat anti-chicken (1:200; Vector, cat no. BA-9010. RRID:AB_2336114) and pacific blue streptavidin (1:200; Thermo Scientific, cat no. S11222). For RAE1 double labeling with β-tubulin III and STMN2, primary and secondary labeling was performed sequentially (RAE1 first) for each antibody with a repeat blocking stage between. Fluorescence images (1024 × 1024) were acquired sequentially on a laser-scanning confocal microscope (LSM 700 Zeiss) with Zen 2012 software (v8.1 SP1, Zeiss). Coverslips were imaged in a single z section. Tissue sections were mounted with DAPI-containing hard-setting aqueous mounting medium (Vectorsheild, Vector Laboratories) and imaged as a stack of 4-5 × 2μm z sections and exported as a maximum intensity projection TIFF.

#### *In situ* hybridization

Male C57BL/6 mice (aged 7-8 weeks) received a unilateral sciatic nerve partial crush injury as described. On day 6 post-injury mice were perfused with 0.01M PBS followed by 4% PFA in 0.1M PBS buffer. Bilateral lumbar DRG were dissected and post-fixed overnight at 4°C followed by cryopreservation in 30% sucrose in PBS for 5 days at 4°C. DRG tissues were embedded in OCT compound, 10 μm sections were cut on a cryostat and thaw-mounted to Superfrost slides and stored at −80°C. Sections from the ipsilateral and contralateral DRG of each mouse were mounted on the same slide. *In situ* hybridization (ISH) was performed with RNA Scope 2.5 HD assay (Red) (322350) according to the manufacturer’s instructions (Advanced Cell Diagnostics, Bio-Techne). Slides were allowed to reach room temperature (RT) then washed once in PBS to remove OCT compound followed by hydrogen peroxide treatment 10 min at RT. Sections the underwent antigen retrieval by submerging in boiling retrieval buffer for 2 min followed by wash in distilled water. After ethanol (100%) treatment sections were treated with Protease Plus reagent (ACD) for 30 min at 40°C in a humidified chamber. Slides were then incubated with probe Mm-Raet1 (448121; targets all *Raet1* isoforms) or positive and negative control probes for 2 h at 40°C. Following thorough washes sections were incubated with six rounds of amplification reagents followed by signal development with fast red. Sections were then processed for NeuN immunohistochemistry. Briefly, sections were blocked with 10% normal goat serum, 0.3% triton-x in 0.01M PBS (1 h, RT) followed by incubation with a chicken anti-NeuN antibody (1:400) overnight at 4°C. The next day sections underwent sequential labeling with biotinylated goat anti-chicken antibody (1:200) and pacific blue-conjugated streptavidin (1:200).

#### Transmission electron microscopy

On day 6 after partial crush mice were perfused with 0.1 M sodium cacodylate trihydrate and 0.1 M sucrose buffer, pH 7.3, followed by 4% paraformaldehyde and 2.5% glutaraldehyde (EM grade) in 0.1 M sodium cacodylate buffer, pH 7.3. Bilateral sciatic nerves were dissected and post-fixed in same fixative and washed three times in 0.1 M sodium cacodylate (SC) buffer only. Samples were carefully trimmed down in a proximal direction toward the crush site. Samples were washed 1x 20 min with 50mM glycine in 0.1M SC buffer with rotation followed by 1x 10-20 min with 0.1M SC buffer. Staining was performed with 1.5% potassium ferrocyanide + 1.5% osmium in 0.1M SC buffer for 4hrs at 4°C with vigorous rotation followed by wash 3x 10 min and 1x30 min with MQ water. Finally, sampled were stained with 0.5% uranyl acetate overnight at 4°C and washed 3x 60 min with water. The dehydration and resin infiltration was performed in the Leica AMW microwave. Briefly, this consisted of 1-2min steps in each 30%, 50%, 70%, 90%, 95% and 100% ethanol at 37°C with constant 25W power, followed by 100% acetone with 3x changes over 5 min. Resin infiltration was performed with 3:1, 1:1, 1:3 acetone:epoxy resin (Ducrupan) in 3 min steps at 37°C and 10W. Samples were then taken through 5x changes 100% resin over 80 mins at 45°C, 12 W, with the final resin step performed at 50°C. Nerves were then embedded in fresh Durcupan resin in flat dish molds at 60°C for 48 hr. Semithin (500nm) and ultrathin sections (90nm) were taken approximately 400 μm in from the proximal end of the crush site of each nerve. Semithin sections were transferred to glass slides and stained with Toludine blue. Ultrathins were transferred to formvar coated 50 mesh copper grids and post-stained for 5 mins with lead citrate. Grids were imaged at 120 kV in a FEI Tecnai 12 TEM using a Gatan OneView camera.

Myelinated axons were classified as follows: *Normal* - Uniform compact myelin sheath with continuous axoplasm; *Abnormal* - Axons surrounded by myelin abnormalities including tomaculae, myelin out-foldings and recurrent myelin loops (Goebbels et al., 2012); *Degenerated* - Absent or vacuous axoplasm surrounded by myelin debris. The number of each axon category per field of view (1471 μm^2^) was counted by an observer blind to the treatment group.

#### Western blot

Tissues and cells were homogenized in RIPA buffer (Millipore, Cat# 20-188) containing protease inhibitor cocktail (Sigma, P8340) and phosphatase inhibitor cocktail (Gendepot, Cat# P3200). Cultured cells were washed with warmed HBSS and collected in protein lysis buffer by scraping; frozen tissues were disrupted either in a Minilys bead homogenizer (Precellys, Bertin, France) or glass grinder. Homogenized samples were then sonicated (3 × 10 s, 25% amplitude) on ice and then spun at high speed (10,000 g) for 10 min at 4°C after 40 min incubation on ice and pellet discarded. An equal volume of 5X SDS sample buffer was added to the sample lysates which were boiled at 9°C heat block for 5 min. Protein content was determined by colorimetric assay (Lowry, BioRad). Equal amounts of protein (25-40 μg) and protein size markers were separated by SDS-polyacrylamide gel electrophoresis (5% stacking gel, 10% resolving gel) followed by transfer to a PVDF membrane. Membranes were blocked in a 5% skimmed milk solution containing Tris buffered saline and 0.1% tween-20 (TBS-T) at room temperature for 1h and subsequently incubated with goat anti-mouse Pan-RAE1 antibody (1:500; R&D systems, cat no. AF1136. RRID:AB_2238016) overnight at 4°C in blocking solution. Blots were washed with TBS-T (3 × 10 min) and then incubated with anti-goat HRP-conjugated secondary antibody (1:10,000; Santa Cruz, cat no. sc-2020. RRID:AB_631728) for 1 h at room temperature. After washing with TBS-T blots were developed by application of western ECL substrate (BioRad, cat no. 1705061) according to the manufacturer’s instructions and images of sequential exposure times were acquired digitally (ChemiDoc, BioRad). Blots were then stripped with stripping buffer at 50°C for 30 min followed by TBS-T washing and subsequently incubated with mouse anti-beta-actin (1:10,000; Sigma, cat no. A5441. RRID:AB_476744) and rabbit anti-N-cadherin (1:10,000; Millipore, cat no. 04-1126. RRID:AB_1977064) in blocking solution. Goat anti-mouse polyclonal (1:10.000; Komabiotech, cat no. K-0211589. RRID:AB_2636911) or goat anti-rabbit (1:10,000; Santa Cruz, cat no. sc-2004. RRID:AB_631746) HRP-conjugated secondary antibodies were applied, respectively, and then exposed after ECL treatment. Embryonic mouse head tissue was used as positive control for RAE1.

For RAE1 expression in tightly ligated sciatic nerve 7 days after injury, mice were perfused with PBS and ipsilateral sciatic nerve was cut into two 5 mm sections: one flanking the crush site and one proximal to the spinal nerves. Tissues were snap frozen in liquid nitrogen and stored at −80°C for later analysis.

#### Enzyme-linked immunosorbant assay (ELISA)

Granzyme B content of sciatic nerves was determined by ELISA according to the manufacturer’s instructions (DuoSet, R&D Systems, cat no. DY1865). Briefly, 96 well plates were coated with capture antibody overnight at RT, washed and blocked 1h. Frozen tissues were homogenized on a shaker with 1.4 mm zirconium beads (Precellys, Bertin) in RIPA lysis buffer (Millipore) and lysates were centrifuged 13,000 rpm for 5 min. Supernatants diluted 1:1 with reagent diluent, added in duplicate to coated wells along with a granzyme B standard series and incubated at RT for 2 h. Plates were washed thoroughly before addition of Streptavidin-HRP, followed by substrate buffer. Stop solution was applied and absorbance (450 nm minus 540 nm correction) read on a microplate spectrophotometer (BioTek Instruments, VT, US). Granzyme B levels in samples were analyzed using the four parameter logistic curve fitting algorithm by reference to standard curve values and performed using online software (https://elisaanalysis.com/).

#### Reverse transcription polymerase chain reaction (RT-PCR)

Total RNA was extracted from L5 DRG from L5x or sham mice (two mice pooled per sample) and L3-L5 DRG from sciatic crush or sham mice (one mouse per sample). Tissues were disrupted in RTL Plus lysis buffer (QIAGEN) including 1% β-mercaptoethanol with a mini glass mortar and pestle on ice and further homogenized using a Minilys bead homogenizer (Precellys, Bertin, France); DRG cultures were washed once in warm HBSS prior to lysis by pipetting; frozen cell pellets were vortexed in lysis buffer. RNA was purified from lysed samples, including genomic DNA elimination, by on-column extraction (RNeasy Plus, QIAGEN) according to the manufacturer’s instructions. RNA was eluted in RNase-free water and analyzed for purity (260/280 nm ratios of approximately 2.0 were considered acceptable) and nucleotide content on a spectrophotometer. Equal amounts of RNA (150-250 ng from whole DRG, 500ng from cell cultures) was reversed-transcribed using M-MLV (200 U/rxn), dNTPs and oligo(dT)_12-18_ primers (Invitrogen) in a 20 μl reaction volume according to the manufacturer’s instructions. PCR reactions (25 μl) using Go Taq Flexi DNA polymerase (Promega) were then performed from cDNA on a thermal cycler (MJ Mini, BioRad) with the following primer pairs: *Raet1a-e* universal primers (*Raet1 alpha*, GenBank: NM_009016.1; *Raet1 beta*, GenBank: NM_009017.1; *Raet1 gamma*, GenBank: NM_009018.1; *Raet1 delta*, GenBank: NM_020030.2; *Raet1 epsilon*, GenBank: NM_198193.2) (270bp), (forward^∗^) 5′-GCTGTTGCCACAGTCACATC-3′, (reverse) 5′-CCTGGGTCACCTGAAGTCAT-3′; *NK1.1* (*Klrb1c v1,* GenBank: NM_001159904.1; *Klrb1c v2,* GenBank: NM_008527.2) (277bp), (forward^∗^) 5′- GGAACAGAGCAGAGCATTCA-3′, (reverse^∗^) 5′-CCAATCAGGGTCAGGACAAG-3′; *Advillin* (NM_009635.3) (332bp), (forward^∗^) 5′-GCTACATCGTCCTCTCGACC-3′, (reverse^∗^) 5′-CATTTCCACCTCCGTGGCTT-3′; *Gapdh* (*Gapdh v1*, GenBank: NM_001289726.1; *Gapdh v2*, GenBank: NM_008084.3) (230bp), (forward^∗^) 5′-AACAGCAACTCCCACTCTTC-3′, (reverse^∗^) 5′-TGGGTGCAGCGAACTTTAT-3′. Asterisk (^∗^) indicates primers which cross an exon-exon boundary. The PCR conditions were 95°C (2 min), cycled 35 times from 95°C (30 s) to 60°C (30 s) to 72°C (30 s), 72°C (5 min), 4°C (forever). Reactions performed without cDNA served as negative control. PCR products were run on an agarose (1.5%) gel containing a DNA staining reagent (SafePinky, GenDepot, USA) and visualized on a UV transilluminator.

#### Quantitative real-time PCR

Gene expression was analysis performed on cDNA from DRG using a Power SYBR Green PCR Master Mix (Applied Biosystems) and pairs of target-specific primers (500nM) in MicroAmp optical tubes (20μl reaction volume) on a 7500 Real-Time PCR system (Applied Biosystems). The PCR conditions were 50°C (2 min), 95°C (10 min), and cycled 40 times at 95°C (15 s) to 60°C (1 min). Samples were run in triplicate. Data were analyzed using the built-in 7500 software (v2.0.4, Life Technologies) and expression was determined relative to a reference gene (*Gapdh*) in adult DRG for cultures or contralateral DRG for nerve injury experiments using the comparative C_t_ method. Primers were designed using Primer-BLAST software (NIH). Primers were selected based on specificity to the desired target gene upon BLAST search, overlap of the exon-exon boundary, lack of potential hairpin-forming or self-priming regions, single peak in the dissociation curve (single band PCR product) and equivalent amplification efficiency (linear shift in C_t_ value upon serial dilution of cDNA). Products of reverse transcription reactions omitting RNA or M-MLV (-RT) served as negative controls. Primer pairs were synthesized by a commercial supplier (Bionics, Korea) with the following sequences: *Raet1a-e* universal primers designed to detect all five *Raet1* isoforms (α,β,γ,δ,ε) (60bp), (forward^∗^) 5′-AACGGGCTGGATGATGCAC-3′, (reverse^∗^) 5′-TGGGGTAGGATCCTTGATGGT-3′; *Gapdh* (72bp), (forward^∗^) 5′-TCCATGACAACTTTGGCATTG-3′, (reverse^∗^) 5′-CAGTCTTCTGGGTGGCAGTGA-3′. Asterisk (^∗^) indicates primers which cross an exon-exon boundary.

### Quantification and Statistical Analysis

#### Laser scanning confocal imaging and analysis

For analysis of neurite density in microfluidic co-cultures, single z section low magnification (x10) confocal images at 0.5 zoom (LSM700, Zeiss) were acquired of β-tubulin III immunofluorescence (647nm emission) along the full length of the neurite compartment. Gamma high channel images were exported as an unaltered TIFF using Zeiss imaging software (v8.1, ZEN 2012 SP1, Zeiss). Images were converted to b/w by adjusting to a set threshold, scale calibrated and horizontal pixel density was measured using the Plot Profile function in ImageJ (v1.46r, NIH). The distance to 50% neurite density was calculated from the normalized cumulative pixel density. 6-8 images per were acquired per microfluidic device, n = 3 devices per group.

For analysis of neurite fragmentation in NK-DRG co-cultures, 5-10 fields of view were randomly selected from each coverslip in a non-systematic manner for acquisition in single or double z section confocal images at low magnification (x20) and 0.5 zoom (LSM700, Zeiss). For total neurite fragmentation gamma high channel images of β-tubulin III immunofluorescence were exported as an unaltered TIFF. In ImageJ images were scale calibrated (1.6 pixels/μm) and brightness threshold set in b/w mode, creating a whole neuron silhouette for selection. Neurite fragments were selected using the *Analyze Particles* function (size 0.5-25 μm^2^, circularity 0-1) and saved as a drawing. The total area and particular area values (μm^2^) were obtained using the *Measure* function. Total DRG cell body area was measured separately and subtracted from the total area to calculate the percent specific neurite fragmentation for each field of view.

For analysis of β-tubulin III and STMN2 labeling in sciatic nerve tissue, a composite of the full length nerve was created from individual z section images acquired at x10 magnification and 0.5 zoom along the length of the nerve (LSM700, Zeiss). Images containing crush site, as well as proximal and distal regions (±1-2 mm from the crush site) were exported to ImageJ, scale calibrated (0.8 pixels/μm) and brightness threshold set in b/w mode. The mean pixel density from a 9000 μm^2^ selection of the nerve mid-image was recorded from 3-4 images per nerve region, per mouse, per treatment.

Co-localized regions of RAE1 double-labeling with β-tubulin III and STMN2 were exported using the co-localization function on the Zen 2012 software after setting for threshold image intensity in both fluorescence channels (1000 units per channel).

For *in situ* hybridization, 3x 2 μm z sections were acquired using x20 apochromat lens at 1x digital zoom. A tile scan of 1024x1024 pixels, 12 bit images was performed with 5% overlap. Image re-stitching was performed offline. Acquisition settings were set to maximize intensity of positive control probe signal while maintaining negligible background on sections labeled with negative control probe. Identical 405 nm and 546 nm channel settings were used throughout imaging of all sections. Images were exported as maximum intensity projection TIFs for image analysis. Images were processed and analyzed in ImageJ. A 500 μm2 sample area of each DRG section was selected and cropped. Blue channel (NeuN) images were used to guide manual selection of individual neuron outlines, which were saved to the region of interest (ROI) manager. ROI selections were then applied to the red channel (*in situ*), which was converted to an 8-bit black/white image with threshold set at 1-255. The number of particles per NeuN+ ROI (larger than 1 μm^2^ to eliminate background spots) were counted using the Particles Analysis function in ImageJ and exported to a spreadsheet (Microsoft Excel). A total of n = 2138 NeuN+ ROI from ipsilateral DRG and n = 1565 NeuN+ ROI from contralateral DRG were included; n = 3 mice, n = 3 sections per DRG per mouse. Data are presented as a frequency distribution of the number of *in situ* spots per neuron (NeuN+ ROI), and versus neuronal cell size (μm^2^).

#### Statistical Analysis

Comparisons between two groups of data were made with Student’s t test (paired or unpaired) or Mann-Whitney test for non-normal distributions (confirmed with Kolmogorov-Smirnov test). Comparisons between three or more groups of data were made with One-way ANOVA. All tests are two-sided unless indicated. Two-way ANOVA tests were used to compare the effects of treatments on molecular, cellular and behavioral assays over time. Data are presented as mean ± standard error of the mean (SEM) unless otherwise stated. *ns*, not significant. Analyses were performed using GraphPad Prism version 5.00 for Windows, GraphPad Software, San Diego California USA, https://www.graphpad.com/. No statistical methods were used to predetermine sample sizes, which were based on previous literature and availability of animals. For behavior tests experimenters were blinded either to the treatment or genotype of the mice during both surgery and sensory testing. Treatments were assigned to littermates at random by an independent observer. p < 0.05 was considered significant.

## Supplemental Information

Table S1. Primer Oligonucleotide Sequences, Related to Figure 1

## References

[bib1] Arapovic J., Lenac T., Antulov R., Polic B., Ruzsics Z., Carayannopoulos L.N., Koszinowski U.H., Krmpotic A., Jonjic S. (2009). Differential susceptibility of RAE-1 isoforms to mouse cytomegalovirus. J. Virol..

[bib2] Backström E., Chambers B.J., Kristensson K., Ljunggren H.G. (2000). Direct NK cell-mediated lysis of syngenic dorsal root ganglia neurons in vitro. J. Immunol..

[bib3] Backström E., Chambers B.J., Ho E.L., Naidenko O.V., Mariotti R., Fremont D.H., Yokoyama W.M., Kristensson K., Ljunggren H.G. (2003). Natural killer cell-mediated lysis of dorsal root ganglia neurons via RAE1/NKG2D interactions. Eur. J. Immunol..

[bib4] Backström E., Ljunggren H.G., Kristensson K. (2007). NK cell-mediated destruction of influenza A virus-infected peripheral but not central neurones. Scand. J. Immunol..

[bib5] Bennett G.J., Doyle T., Salvemini D. (2014). Mitotoxicity in distal symmetrical sensory peripheral neuropathies. Nat. Rev. Neurol..

[bib6] Boyman O., Kovar M., Rubinstein M.P., Surh C.D., Sprent J. (2006). Selective stimulation of T cell subsets with antibody-cytokine immune complexes. Science.

[bib7] Bridge P.M., Ball D.J., Mackinnon S.E., Nakao Y., Brandt K., Hunter D.A., Hertl C. (1994). Nerve crush injuries--a model for axonotmesis. Exp. Neurol..

[bib8] Cerwenka A., Bakker A.B., McClanahan T., Wagner J., Wu J., Phillips J.H., Lanier L.L. (2000). Retinoic acid early inducible genes define a ligand family for the activating NKG2D receptor in mice. Immunity.

[bib9] Chandran V., Coppola G., Nawabi H., Omura T., Versano R., Huebner E.A., Zhang A., Costigan M., Yekkirala A., Barrett L. (2016). A systems-level analysis of the peripheral nerve intrinsic axonal growth program. Neuron.

[bib10] Chavan S.S., Pavlov V.A., Tracey K.J. (2017). Mechanisms and therapeutic relevance of neuro-immune communication. Immunity.

[bib11] Cho Y., Sloutsky R., Naegle K.M., Cavalli V. (2013). Injury-induced HDAC5 nuclear export is essential for axon regeneration. Cell.

[bib12] Conforti L., Gilley J., Coleman M.P. (2014). Wallerian degeneration: An emerging axon death pathway linking injury and disease. Nat. Rev. Neurosci..

[bib13] Costigan M., Scholz J., Woolf C.J. (2009). Neuropathic pain: A maladaptive response of the nervous system to damage. Annu. Rev. Neurosci..

[bib14] Cui J.G., Holmin S., Mathiesen T., Meyerson B.A., Linderoth B. (2000). Possible role of inflammatory mediators in tactile hypersensitivity in rat models of mononeuropathy. Pain.

[bib15] Cusack C.L., Swahari V., Hampton Henley W., Michael Ramsey J., Deshmukh M. (2013). Distinct pathways mediate axon degeneration during apoptosis and axon-specific pruning. Nat. Commun..

[bib16] Dekkers M.P., Nikoletopoulou V., Barde Y.A. (2013). Cell biology in neuroscience: Death of developing neurons: New insights and implications for connectivity. J. Cell Biol..

[bib17] Duvdevani R., Rosner M., Belkin M., Sautter J., Sabel B.A., Schwartz M. (1990). Graded crush of the rat optic nerve as a brain injury model: Combining electrophysiological and behavioral outcome. Restor. Neurol. Neurosci..

[bib18] Dyck P.J., Hopkins A.P. (1972). Electron microscopic observations on degeneration and regeneration of unmyelinated fibres. Brain.

[bib19] Gamage K.K., Cheng I., Park R.E., Karim M.S., Edamura K., Hughes C., Spano A.J., Erisir A., Deppmann C.D. (2017). Death Receptor 6 Promotes Wallerian Degeneration in Peripheral Axons. Curr. Biol..

[bib20] Garrod K.R., Wei S.H., Parker I., Cahalan M.D. (2007). Natural killer cells actively patrol peripheral lymph nodes forming stable conjugates to eliminate MHC-mismatched targets. Proc. Natl. Acad. Sci. USA.

[bib21] Gaudet A.D., Popovich P.G., Ramer M.S. (2011). Wallerian degeneration: Gaining perspective on inflammatory events after peripheral nerve injury. J. Neuroinflammation.

[bib22] Gerdts J., Summers D.W., Milbrandt J., DiAntonio A. (2016). Axon self-destruction: New links among SARM1, MAPKs, and NAD+ metabolism. Neuron.

[bib23] Goebbels S., Oltrogge J.H., Wolfer S., Wieser G.L., Nientiedt T., Pieper A., Ruhwedel T., Groszer M., Sereda M.W., Nave K.A. (2012). Genetic disruption of Pten in a novel mouse model of tomaculous neuropathy. EMBO Mol. Med..

[bib24] Gonzalez-Cano R., Boivin B., Bullock D., Cornelissen L., Andrews N., Costigan M. (2018). Up-down reader: An open source program for efficiently processing 50% von Frey thresholds. Front. Pharmacol..

[bib25] Greene T.T., Tokuyama M., Knudsen G.M., Kunz M., Lin J., Greninger A.L., DeFilippis V.R., DeRisi J.L., Raulet D.H., Coscoy L. (2016). A Herpesviral induction of RAE-1 NKG2D ligand expression occurs through release of HDAC mediated repression. eLife.

[bib26] Hickey W.F., Ueno K., Hiserodt J.C., Schmidt R.E. (1992). Exogenously-induced, natural killer cell-mediated neuronal killing: A novel pathogenetic mechanism. J. Exp. Med..

[bib27] Lackowski D., Koberda J.L., DeLoughery T.G., So Y. (1998). Natural killer cell leukemia as a cause of peripheral neuropathy and meningitis: Case report. Neurology.

[bib28] Latremoliere A., Cheng L., DeLisle M., Wu C., Chew S., Hutchinson E.B., Sheridan A., Alexandre C., Latremoliere F., Sheu S.H. (2018). Neuronal-specific TUBB3 is not required for normal neuronal function but is essential for timely axon regeneration. Cell Rep..

[bib29] Lopez J.A., Susanto O., Jenkins M.R., Lukoyanova N., Sutton V.R., Law R.H., Johnston A., Bird C.H., Bird P.I., Whisstock J.C. (2013). Perforin forms transient pores on the target cell plasma membrane to facilitate rapid access of granzymes during killer cell attack. Blood.

[bib30] Loux T.J., Lotze M.T., Zeh H.J., Lotze M.T., Thomson A.W. (2010). NK cells as recipients of cytokine signals. Natural Killer Cells: Basic Science and Clinical Application.

[bib31] Mishra S.K., Tisel S.M., Orestes P., Bhangoo S.K., Hoon M.A. (2011). TRPV1-lineage neurons are required for thermal sensation. EMBO J..

[bib32] Morvan M.G., Champsaur M., Reizis B., Lanier L.L. (2017). Chronic in vivo interaction of dendritic cells expressing the ligand Rae-1ε with NK cells impacts NKG2D expression and function. Immunohorizons.

[bib33] Narni-Mancinelli E., Chaix J., Fenis A., Kerdiles Y.M., Yessaad N., Reynders A., Gregoire C., Luche H., Ugolini S., Tomasello E. (2011). Fate mapping analysis of lymphoid cells expressing the NKp46 cell surface receptor. Proc. Natl. Acad. Sci. USA.

[bib34] Neukomm L.J., Freeman M.R. (2014). Diverse cellular and molecular modes of axon degeneration. Trends Cell Biol..

[bib35] Ogasawara K., Hamerman J.A., Ehrlich L.R., Bour-Jordan H., Santamaria P., Bluestone J.A., Lanier L.L. (2004). NKG2D blockade prevents autoimmune diabetes in NOD mice. Immunity.

[bib36] Raulet D.H., Gasser S., Gowen B.G., Deng W., Jung H. (2013). Regulation of ligands for the NKG2D activating receptor. Annu. Rev. Immunol..

[bib37] Ren K., Dubner R. (2010). Interactions between the immune and nervous systems in pain. Nat. Med..

[bib38] Sander S., Ouvrier R.A., McLeod J.G., Nicholson G.A., Pollard J.D. (2000). Clinical syndromes associated with tomacula or myelin swellings in sural nerve biopsies. J. Neurol. Neurosurg. Psychiatry.

[bib39] Sheikh S., Parhar R.S., Bakheet R., Saleh S., Collison K., Al-Mohanna F. (2004). Immobilization of rolling NK cells on platelet-borne P-selectin under flow by proinflammatory stimuli, interleukin-12, and leukotriene B4. J. Leukoc. Biol..

[bib40] Tandrup T., Woolf C.J., Coggeshall R.E. (2000). Delayed loss of small dorsal root ganglion cells after transection of the rat sciatic nerve. J. Comp. Neurol..

[bib41] Tang Y., Peitzsch C., Charoudeh H.N., Cheng M., Chaves P., Jacobsen S.E., Sitnicka E. (2012). Emergence of NK-cell progenitors and functionally competent NK-cell lineage subsets in the early mouse embryo. Blood.

[bib42] Vivier E., Raulet D.H., Moretta A., Caligiuri M.A., Zitvogel L., Lanier L.L., Yokoyama W.M., Ugolini S. (2011). Innate or adaptive immunity? The example of natural killer cells. Science.

[bib43] Voskoboinik I., Whisstock J.C., Trapani J.A. (2015). Perforin and granzymes: Function, dysfunction and human pathology. Nat. Rev. Immunol..

[bib44] Votavova P., Tomala J., Kovar M. (2014). Increasing the biological activity of IL-2 and IL-15 through complexing with anti-IL-2 mAbs and IL-15Rα-Fc chimera. Immunol. Lett..

[bib45] Wang J.T., Medress Z.A., Barres B.A. (2012). Axon degeneration: Molecular mechanisms of a self-destruction pathway. J. Cell Biol..

[bib46] Williams P.R., Marincu B.N., Sorbara C.D., Mahler C.F., Schumacher A.M., Griesbeck O., Kerschensteiner M., Misgeld T. (2014). A recoverable state of axon injury persists for hours after spinal cord contusion in vivo. Nat. Commun..

[bib47] Willis D., Li K.W., Zheng J.Q., Chang J.H., Smit A.B., Kelly T., Merianda T.T., Sylvester J., van Minnen J., Twiss J.L. (2005). Differential transport and local translation of cytoskeletal, injury-response, and neurodegeneration protein mRNAs in axons. J. Neurosci..

[bib48] Xie W., Strong J.A., Zhang J.M. (2017). Active nerve regeneration with failed target reinnervation drives persistent neuropathic pain. eNeuro.

[bib49] Ziegler E.A., Magerl W., Meyer R.A., Treede R.D. (1999). Secondary hyperalgesia to punctate mechanical stimuli. Central sensitization to A-fibre nociceptor input. Brain.

