# Supplementary material for: Natural Killer Cells Degenerate Intact Sensory Afferents following Nerve Injury
Source: Cell. 2019 Feb 7;176(4):716–728.e18. doi: 10.1016/j.cell.2018.12.022 (PMC6418410; doi:10.1016/j.cell.2018.12.022)
Supplement: Table S1. Primer Oligonucleotide Sequences, Related to Figure 1 [file mmc1.pdf]

Cell, Volume *176*

Supplemental Information

Natural Killer Cells Degenerate Intact Sensory Afferents following Nerve Injury

Alexander J. Davies, Hyoung Woo Kim, Rafael Gonzalez-Cano, Jahyang Choi, Seung Keun Back, Seung Eon Roh, Errin Johnson, Melanie Gabriac, Mi-Sun Kim, Jaehee Lee, Jeong Eun Lee, Yun Sook Kim, Yong Chul Bae, Sang Jeong Kim, Kyung-Mi Lee, Heung Sik Na, Priscilla Riva, Alban Latremoliere, Simon Rinaldi, Sophie Ugolini, Michael Costigan, and Seog Bae Oh

Table S1. **Related to Figure 1.** Primer oligonucleotide sequences.

**Primers**	**Forward sequence**	**Reverse sequence**
PCR: *Raet1a-e*	GCTGTTGCCACAGTCACATC	CCTGGGTCACCTGAAGTCAT
PCR: *NK1.1*	GGAACAGAGCAGAGCATTCA	CCAATCAGGGTCAGGACAAG
PCR: *Advillin*	GCTACATCGTCCTCTCGACC	CATTTCCACCTCCGTGGCTT
PCR: *Gapdh*	AACAGCAACTCCCACTCTTC	TGGGTGCAGCGAACTTTAT
Real time: *Raet1a-e*	AACGGGCTGGATGATGCAC	TGGGGTAGGATCCTTGATGGT
Real time: *Gapdh*	TCCATGACAACTTTGGCATTG	CAGTCTTCTGGGTGGCAGTGA
